# Cross-sectional Study of the Burden of Vector-Borne and Soil-Transmitted Polyparasitism in Rural Communities of Coast Province, Kenya

**DOI:** 10.1371/journal.pntd.0002992

**Published:** 2014-07-24

**Authors:** Donal Bisanzio, Francis Mutuku, Amaya L. Bustinduy, Peter L. Mungai, Eric M. Muchiri, Charles H. King, Uriel Kitron

**Affiliations:** 1 Department of Environmental Sciences, Emory University, Atlanta, Georgia, United States of America; 2 Department of Environment and Health Sciences, Technical University of Mombasa, Mombasa, Kenya; 3 Parasitology Department, Liverpool School of Tropical Medicine, Liverpool, United Kingdom; 4 Center for Global Health and Diseases, Case Western Reserve University, Cleveland, Ohio, United States of America; 5 Division of Vector-Borne and Neglected Tropical Diseases, Ministry of Public Health and Sanitation, Nairobi, Kenya; Universidad San Francisco de Quito, Ecuador

## Abstract

**Background:**

In coastal Kenya, infection of human populations by a variety of parasites often results in co-infection or poly-parasitism. These parasitic infections, separately and in conjunction, are a major cause of chronic clinical and sub-clinical human disease and exert a long-term toll on economic welfare of affected populations. Risk factors for these infections are often shared and overlap in space, resulting in interrelated patterns of transmission that need to be considered at different spatial scales. Integration of novel quantitative tools and qualitative approaches is needed to analyze transmission dynamics and design effective interventions.

**Methodology:**

Our study was focused on detecting spatial and demographic patterns of single- and co-infection in six villages in coastal Kenya. Individual and household level data were acquired using cross-sectional, socio-economic, and entomological surveys. Generalized additive models (GAMs and GAMMs) were applied to determine risk factors for infection and co-infections. Spatial analysis techniques were used to detect local clusters of single and multiple infections.

**Principal findings:**

Of the 5,713 tested individuals, more than 50% were infected with at least one parasite and nearly 20% showed co-infections. Infections with *Schistosoma haematobium* (26.0%) and hookworm (21.4%) were most common, as was co-infection by both (6.3%). Single and co-infections shared similar environmental and socio-demographic risk factors. The prevalence of single and multiple infections was heterogeneous among and within communities. Clusters of single and co-infections were detected in each village, often spatially overlapped, and were associated with lower SES and household crowding.

**Conclusion:**

Parasitic infections and co-infections are widespread in coastal Kenya, and their distributions are heterogeneous across landscapes, but inter-related. We highlighted how shared risk factors are associated with high prevalence of single infections and can result in spatial clustering of co-infections. Spatial heterogeneity and synergistic risk factors for polyparasitism need to be considered when designing surveillance and intervention strategies.

## Author Summary

In Coast Province, Kenya, infections with *Schistosoma haematobium*, *Plasmodium* spp., filarial nematodes, and geohelminths are common, resulting in high levels of both single infections and polyparasitism. The long-term effect of these infections, separately or in combination, has a major impact on human health and on the economic welfare of affected populations. The transmission dynamics of these parasitic infections can be linked to shared risk factors that often overlap in space. We studied human and environmental factors driving transmission and the resulting spatial pattern of infections in six communities, using cross-sectional, socio-economic and entomological surveys. Single and co-infections were widespread in the communities, and were associated with environmental, demographic and socio-economic risk factors, including distance of community from the coast, sanitation and human age and crowding. The spatial patterns of single and co-infections were heterogeneous among and within communities, with overlapping clusters of single and multiple infections in areas where houses with lower socio-economic status and more crowding were located. The heterogeneities among and within communities can provide important insights when designing surveillance and intervention strategies when planning appropriate surveillance and control strategies targeting polyparasitism.

## Introduction

In coastal Kenya, multiple parasite species infect human populations and their transmission dynamics can significantly overlap. In this ecological setting, transmission of *Schistosoma haematobium*, *Plasmodium* spp., filarial nematodes, and geohelminths is common, resulting in high levels of concurrent human urinary schistosomiasis, malaria, hookworm infection and/or ascariasis, as well as pockets of lymphatic filariasis [1], [2], [3], [4], [5]. Because of their combined long-term effects, these infections appear to play a significant but, as yet, incompletely defined synergistic role in the causation of chronic clinical and sub-clinical human disease and poverty [6], [7], [8]. In this context, transmission patterns and risk factors for these diverse parasitic infections often appear to be linked and to overlap extensively [9], [10], [11]. We hypothesized that people living in areas where environmental factors allow for coincident transmission of several parasites would have a much higher chance of suffering from multiple concurrent infections. Although the interaction between parasites [12], [13] is still not fully understood, now in the era of integrated parasite control programs, it is important to define those factors that enhance risk of co-infection. This challenge has been approached by several studies that investigated the complexity of multi-parasite ecology, focusing on heterogeneities in infection risk across physical and social space, and over time [12], [14], [15], [16], [17], [18].

Building on our earlier studies of schistosomiasis, we hypothesized that environmental factors are the key determinants of transmission potential for these parasites, and that these interact with demographic and socio-economic factors to determine the observed spatial/demographic patterns of parasitic disease. While this in itself is not a new concept [19], recent research on parasite eco-epidemiology indicate that these effects need to be reconsidered on multiple levels–individual, household, village, and district-wide– both separately for each parasite, and for the combined suite of infections [18], [20], [21], [22].

Although ‘*wormy villages*’ have been described empirically in the past [23], new advances in diagnostic technology have increased test sensitivity and specificity for these parasites, revealing that in endemic areas, chronic parasitic infection with *Schistosoma* spp. [24], *Plasmodia* spp. [25], and/or filaria [26] are much more common than previously thought. In holoendemic areas such as coastal Kenya or Papua New Guinea, malaria prevalence, as detected by PCR is 60–75%, more than double the previous estimates of 20–33% by blood smear microscopy, with ≥10% carrying two or more malaria species [25], [27]. This finding dramatically changes our concept of malaria as a chronically prevalent disease, and substantially alters estimates of attributable risk for critical infection-associated morbidities such as anemia [28]. Similarly, advances in filaria antigen detection techniques indicate that past community surveys have underestimated prevalence of filariasis by 40% [29], while standard screening techniques for *S. haematobium* have probably missed 50–60% of low level infections with this parasite [30]. These findings indicate the need to carefully re-evaluate the risk of infection and parasite-related morbidity in exposed populations.

The role of the environment is assumed to be critical for vector-borne and soil-transmitted parasite transmission, although the relative non-linear impact of individual environmental factors has not been well-quantified [31], [32]. In contrast to person-to-person contagion of viruses and bacteria, there is a difference between a person's exposure to parasites and her or his ultimate level of parasite infections and related diseases, which is often governed by continued residence in the high- risk environment.

Previous studies performed in sub-Saharan (or tropical) countries [9], [16], [33] have pointed to the need for adopt novel quantitative approaches that take into account the issue of scale when investigating the interactions of physical and social space with the risk for poly-parasitism. Our study's aim was to detect spatial and demographic patterns of transmission and infection for schistosomiasis, malaria, filariasis, and soil-transmitted helminths (STH) in coastal Kenya through integration of parasitological data with landscape, land use, and socioeconomic risk factors. Our project is one of the few studies which use socio-ecological data and spatial analysis techniques to examine a large spectrum of co-infections affecting people living in coastal Kenya. By combining remotely sensed and directly measured environmental factors with new aspects of social geography, along with implementation of new diagnostics methods and the use of advanced statistical tools, our analysis provides new insights into polyparasitism that can inform the design and application of more effective, population-based control strategies.

## Methods

### Ethical approval

Ethical approval and oversight for this study was jointly provided by the Institutional Review Board of the University Hospital Case Medical Center of Cleveland (Protocol 11-07-45) and by the Ethical Review Committee of the Kenya Medical Research Institute (KEMRI) (Non-SSC Protocol 087). All residents of the study villages were eligible for inclusion as participants in the study if they were permanent residents of the selected study communities, and aged 5 years or above. Written informed consent was obtained from the subject or, for minors, his or her parent, prior to participation.

### Populations surveyed

**Figure 1:**
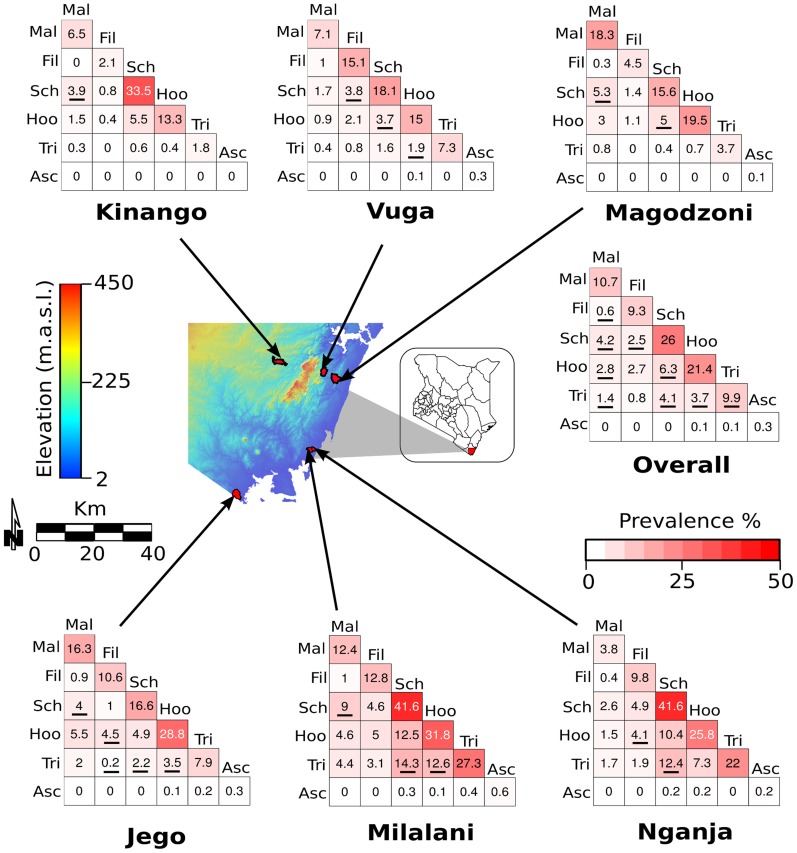
Prevalence of single or co-infections (two parasites) in the study area. The figure shows prevalence of infections at the village level and for all 6 villages together. Values and color intensity in the matrices represent the prevalence of single and each combination of parasite infections. Underlined numbers indicate infection prevalence significantly higher or lower (p<0.05) than expected by random chance (the product of single parasite infection prevalence). The map in the center shows elevation and location of each village. Infection abbreviation: malaria (Mal), filariasis (Fil), schistosomiasis (Sch), hookworm (Hoo), Trichuris (Tri), and Ascaris (Asc).

We conducted this six-village study across four different environmental settings within Kwale County, Coast Province, Kenya during 2009–2011 ([34], [35], [36], [37]). Village selection was aimed at creating a stratified sample of different environments across the County, covering an estimated population of 12,000 people. The required study size was estimated based on the likely prevalence of major co-infections in the area, as reported in previous smaller surveys [5], [38]. The ecological settings were: a. estuary (Jego), b. coastal plain (Magodzoni, Nganja, and Milalani), c. coastal slope (Vuga), and d. inland semi-arid (Kinango) areas (Figure 1, Tables 1). In terms of its demographics and developmental metrics, Kwale County is representative of other rural districts of Kenya (and sub-Saharan Africa) that are burdened by polyparasitism [39]. To optimize participation and limit participation bias, each village survey included preliminary informational meetings, followed by demographic census, including household location by GPS or remotely sensed visual imaging as detailed in our previous schistosomiasis study in Msambweni [40], and their enumeration. At each household, an adult informant was interviewed on household SES using an established, validated questionnaire administered in the local languages (Kidigo or Kiswahili) [36], [41], [42].

**Table 1:** Demographic data and environmental characteristics of each village.

	Jego	Kinango	Magodzoni	Milalani	Nganja	Vuga
*Parasite survey:*						
Period	Apr–May 2010	Oct–Nov 2010	Apr–May 2011	Jul–Aug 2009	Apr–May 2009	Nov–Dec 2009
Number of houses	398	580	381	273	148	384
Number of tested houses^a^	334	390	229	238	148	381
Total population	2750	2641	1860	1645	816	2362
Eligible residents^b^	2351	2089	1632	1437	718	2042
Number of participants tested	1179	1155	737	776	531	1335
Participation rate	50%	55%	45%	54%	74%	65%
Male (%)	48.7	57.7	49.6	48.5	50.6	48.4
Age (%):						
0–5	14.5	16.4	16.5	10.4	8.8	10.8
6–13	26.8	26.4	25.2	23.1	22.7	23.5
14–17	10.7	9.5	9.6	11.4	11.0	13.3
18–21	8.5	5.8	8.2	8.7	9.4	9.9
>21	39.5	41.9	40.4	46.5	47.4	42.5
Adult M∶F ratio^c^	0.55	0.37	0.53	0.65	0.72	0.63
Educated (%)^d^	43.5	44.7	43.1	45.2	38.2	50.1
*Mosquito survey:*						
Number of houses	99	93	74	58	82	72
Number of people	380	329	264	196	290	380
*Environmental*						
*factors:*						
Elevation (mean m.a.s.l.)	15.1	186.2	82.3	24.9	19.1	147.5
Distance to Coast (mean km)	3.1	31.2	6.5	2.7	1.7	12.3
Tree coverage (% of village area)	13.5	13.5	7.4	14.4	18.0	16.9
Rainfall (mm per year)	478.4	212.6	251.1	354.9	354.9	251.1
Temperature (C°, annual mean)	27.4	25.7	26.3	27.2	27.3	26.8

Number of houses and people included in the poly-parasitism survey, and demographic characteristics of each community. The table includes environmental features of each village.

a Houses in which at least one resident was sampled for parasite screening.

b Long term residents 5 years or older;

c Adults 18 years or older;

d Only primary and secondary education.

### Parasitological evaluation

Consenting participants were tested for infection exposure, current infection, and current infection intensity as follows: Current malaria infection was detected initially by rapid antigen-detection card technique (ICT Diagnostics, Australia), and later confirmed and quantified by PCR [27]. In our analysis, an ICT-positive status was the basis for assigning malaria infection; hookworm, *Trichuris*, and *Ascaris* infections were detected and quantified by standard Kato-Katz stool examination (two duplicate smears) of a single stool specimen [43]; The presence of *Wuchereria bancrofti* infection (lymphatic filariasis, LF) was detected by circulating antigen detection (Binax, Portland, ME); *S. haematobium* infection was detected and quantified by Nuclepore urine filtration technique from a single midday urine [44], [45]. Infected subjects received standard anti-parasite treatments at the time of the survey according to the Ministry of Health guidelines.

### Mosquito trapping and surveillance

Mosquito trapping was performed longitudinally over four years (April 2009–April 2013) in all eight study villages. For the period of April 2009 to December 2010, mosquito collections were performed once every 4 weeks using three different methods: Pyrethrum Spray Catch (PSC), Clay pots and Prokopack aspirator. Indoor collections by PSC were performed from April 2009 through December 2010 in 10 randomly selected houses, while outdoor collections by clay pots were performed from April 2009 through August 2010 in 10 randomly selected houses (discontinued due to poor catch).

Mosquito collections using Prokopack aspirator [46] were started in March 2010 and continued through December 2010 in 5 randomly selected houses. Mosquito collections from January 2011 to March 2011 were inconsistent, with only 7 mosquito collection efforts conducted out of the possible 24 for both PSC and Prokopack aspirator. No mosquito collections were performed in January and most of February 2011 due to logistical difficulties. For the period of April 2011 to April 2013, mosquito collections were performed once every 8 weeks in all the eight study villages using PSC and Prokopack aspirator in 10 randomly selected houses for each mosquito collection method.

Mosquito collection by all methods always started at 06:00 h and ended no later than 10:00 h. For PSC catches of indoor resting mosquitoes, houses were sprayed with 10% pyrethrins dissolved in kerosene using the method described by Mutuku and others [34]. Mosquito collection using clay pots and Prokopack aspirator were performed as described by Maia and others [47] and Odiere and others [48].

### SES evaluation

We evaluated the socio-economic standing (SES) of each individual and assigned an SES score based on a set of factors related to asset ownership and the physical characteristics of their home. We considered variables related to ownership of land, house, and durable assets (e.g., radio, motor vehicle, television) (Table 2). Given the heterogeneity among the studied communities in the range of these factors, we adjusted the SES scale for each village. Economic inequity was also estimated based on house characteristics (e.g., number of rooms used for sleeping and building materials) and on access to utilities and infrastructure (e.g. sanitation facility and source of water).

**Table 2:** Entomological collections at the village level.

	Jego	Kinango	Magodzoni	Milalani	Nganja	Vuga
*Entomology* *						
*An. gambiae*	1.5 (0.9–2.4) [32%]	0.07 (0.02–0.18) [5.1%]	0.1 (0.06–0.33) [7.4%]	0.05 (0.01–0.25) [3.5%]	0.01 (4.3×10^−5^–0.03) [1.1%]	0
*An. funestus*	0.8 (0.5–1.2) [29.7%]	0.03 (0.01–0.07) [3.4%]	0.8 (0.5–1.5) [24.7%]	0.4 (0.1–1.3) [10.3%]	0	0.7 (0.2–3.7) [8.8%]
*Culex.* spp	9.5 (7.2–12.8) [75.7%]	19.5 (15–25.9) [82.3%]	4.2 (2.7–6.8) [42.9%]	20.4 (15.7–27.3) [93.1%]	25.3 (19.3–34.1) [91.4%]	16.5 (12.7–21.9) [86.6%]

*Mean number of collected female mosquito per house (95% CI) [% of houses positive for mosquito presence].

The SES score was calculated using Multi Correspondence Analysis (MCA) [49]. MCA is a multivariate method developed for exploring datasets with discrete quantitative values that can be used to create a weight index based on the variance explained by each included variable. The MCA weight index is similar to the one calculated using principal component analysis (PCA) [49], both using a set of linear combinations to account for variability in the data. While PCA is based on variance-covariance matrix, MCA uses a scaling of the Pearson's chi-squared statistic [50]. In calculating the SES scores, we only considered the first MCA linear combination that explained the greater part of the data's variability, then used this score to categorize households of each village into four ordinal groups (Poorest, Poor, Rich, Richest), based on quartiles.

### Statistical modeling

A set of logistic regressions based on generalized additive models (GAMs) [51] was created to analyze the effect of demographic variables, SES, village setting, use of bednets, and entomological measures on presence or absence of parasite infection in the surveyed populations. We also performed a generalized additive mixed model (GAMM) [51] to calculate how these variables were associated with individual co-prevalence of two or more infections. Because we used the number of co-occurring infections of different parasites (poly-parasitism), the GAMM was performed based on a Poisson distribution and, to account for data over-dispersion, individual ID was entered as random effect [52]. In both GAMs and GAMMs, age was included as a non-linear predictor represented by a smooth function [51].

In addition to analyzing the aggregate data for the six villages, we also performed the same modeling analyses for the village of Milalani, the most heavily parasitized village and the one with the highest prevalence of poly-parasitism.

Given the presence of clusters of single and multiple infections, we tested (using Moran's I) whether the same spatial autocorrelation pattern persisted in model residuals, which would indicate a spatial bias, as applied by Dormann et al, 2007 [53].

### Spatial analysis

The spatial patterning of prevalence of individual parasite infections and of poly-parasitism was quantified with the Getis' Gi*(d) local statistic [54], using the inverse distance as the spatial weight. Significance was evaluated by comparing observed values with values expected under the null hypothesis of complete spatial randomness (based on 999 Monte Carlo permutations of location status). We also applied the Gi*(d) to analyze the spatial clustering of greater household crowding and lower SES [54].

### Other statistical analyses

Fisher's least significant difference (LSD) test [55] was applied to determine significant differences in prevalence of infections, SES, sanitation, sources of drinking water, house structure, and mosquito infestation between villages. Wilcoxon signed-rank test was performed to evaluate difference in mean number of female mosquitoes collected per house between communities. The Spearman's nonparametric correlation coefficient, *ρ*, was applied to test a possible association of spatial co-occurrence of clustering of high prevalence of co-infection with of clustering of lower SES and household crowding. We applied this test at the house level to determine whether households included in co-infection clusters were also part of SES or crowding clusters.

### GIS and statistical tools

All geographic data were stored in a Geographic Information System (GIS) using Quantum GIS (QGIS) software [56] georeferenced using Universal Transverse Mercator (UTM) Zone 37 South, datum WGS84. Spatial analysis tests were performed using Easyspat (Bisanzio et al. in prep.), an open-source software based on PySal libraries written in Python language [57]. All other analyses and data cleaning were performed using R software [56].

## Results

### Village environmental characteristics

The environmental characteristics of the six villages are summarized in Table S1 in Text S1. The distance from the coastline ranged from 1.7 to 31.2 Km (Fig. 1). Mean elevation was highest in Kinango (186.2 meters above sea level), and lowest in Jego (15.1 meters). Annual mean temperature and annual rainfall was negatively correlated with distance to the sea and with elevation.

### Village demographic and SES characteristics

Demographic, SES and sanitation attributes are shown in Tables 1 and Table S1 in Text S1 along with participation rates in each village. Participation was incomplete in every village, ranging from 45–74% of eligible residents. Overall, 56% of those eligible completed their full participation in the laboratory testing. Adult female, who are more often at home, had higher rates of participation than adult males (overall M∶F ratio = 0.56). Both of these may have biased our estimates of infection prevalence. Residents of Kinango owned more assets than inhabitants of the other villages. Kinango and Vuga had a significantly higher percentage of houses with both cement floors and iron roofs (Fisher's LSD, p<0.05), and also had a significantly lower number of houses without a sanitation system (Fisher's LSD, p<0.05), and the highest proportion of households with access to a public source of drinking water (Fisher's LSD, p<0.05). Kinango had the highest percentage of households with their own source of drinking water (17.9%, Fisher's LSD, p<0.05).

Average SES was lowest in Jego, which had the lowest proportion of houses with access to sanitation and its inhabitants owned the fewest assets. Milalani, Nganja and Vuga levels of SES, sanitation, and sources of drinking water were intermediate between Jego and Kinango. There was no significant difference in education level between villages (Fisher's LSD, p>0.05).

### Entomology

Entomological data are shown in Table 2. A total of 32,982 female mosquitoes were collected during April 2009–April 2013. *Culex* spp. females were by far the most abundant (31,116; 94.6% of all mosquitoes), followed by *An. gambiae* (988; 3.1%) and *An. funestus* (878; 2.9%). *Culex* spp. mosquitoes also were collected in a significantly higher proportion of households than all other mosquitoes (Table 2, Fisher's LSD, p<0.05).

In Milalani and Nganja, the percentage of houses infested with *Culex* spp. was significantly higher than in the other four villages (Fisher's LSD, p<0.05). The abundance and presence of *Culex* spp. was significantly lower in Magodzoni (Wilcoxon test, Fisher's LSD, p<0.05). *An. funestus* was significantly more abundant in Jego and Magodzoni, and *An. gambiae* was more abundant only in Jego (Table 2, Wilcoxon test, p<0.05; Fisher's LSD, p<0.05).

### Parasitic infections

**Figure 2:**
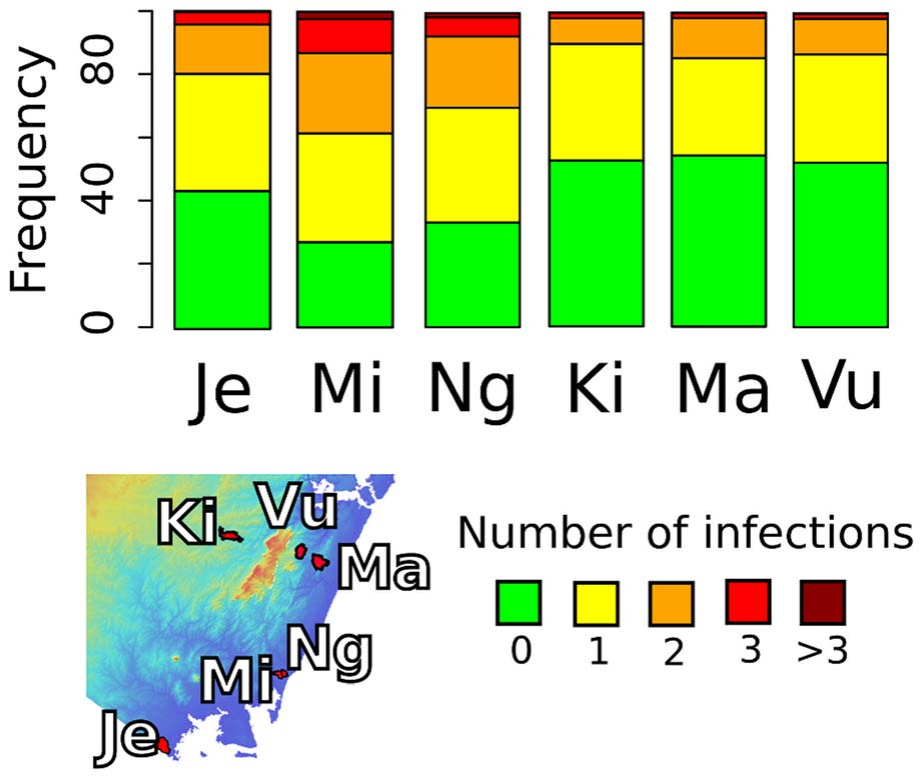
Prevalence of poly-parasitism by village (Je - Jego, Mi - Milalani, Ng - Nganja, Ki - Kinango, Ma - Magodzoni, Vu - Vuga).

The prevalence of infections is presented in Figures 1 and 2 and supplemental Tables S2 and S3 in Text S1. The most common infections among tested individuals were *S. haematobium* (26.0% overall prevalence) and hookworm (21.4%). Co-infection by these two parasites was the most common co-infection (6.3%), significantly more than expected by random chance (the product of single parasite infection prevalences) (Figure 1, Table S2 in Text S1). Prevalence of malaria, filariasis, and *Trichuris* infections were similar and significantly less frequent than *S. haematobium* and hookworm infections (Fisher's LSD, p<0.01). *Ascaris* infection was by far the least common (prevalence of only 0.3%), and was excluded from most of the analyses. Overall, 18.8% of the population was infected by more than one parasite. Two individuals were co-infected by all of the five different parasites.

Prevalence of infections in the six villages was significantly different for all parasites other than the rare *Ascaris*. In Kinango the prevalence of overall parasitic infections was lowest (Fisher's test, p<0.05), with significantly lower prevalence of hookworm and *Trichuris* infections (Fisher's LSD, p<0.01), but not of schistosomiasis. Kinango also had the lowest prevalence of multiple infections. In Vuga, Nganja, and Kinango prevalence of malaria was similar and significantly lower than in the other three villages. People living in Milalani had significantly higher prevalence of infections compared with the other villages (Fisher's LSD, p<0.05). Prevalence of co-infection in Kinango, Vuga, and Magodzoni was significantly lower than the other three villages (Fisher's LSD, p<0.05, Figure 2, Table S3 in Text S1).

### Covariates associated with infection and multiple infections

For the aggregated data from all six villages, results from the GAMs and GAMMs (Table 3), showed that demographic factors (age, gender, and education), SES, and household characteristics (construction, use of bednets, water source, sanitation, and number of inhabitants) were significantly associated with parasite infections and co-infections. Males had a higher risk of being infected by all parasites other than *Schistosoma*, for which gender did not have a significant effect. Males were also more likely to be infected with more than one parasite species. Lower SES, lack of access to sanitation, and absence of a safe source of drinking water were associated with a higher risk of infection and co-infection, although SES was not associated with filariasis or *Trichuris* infection.

**Table 3:** Predictor estimates obtained by GAMs and GAMMs for each single infection and for co-infections.

Predictor	Malaria^b^	Filariasis^b^	Schistosomiasis^b^	Hookworm^b^	Trichuris^b^	Number of co-infections^c^
Sex: Male	**1.32 (0.13)** **	**1.26 (0.14)** *	1.08 (0.08)	**1.65 (0.12)** **	**1.26 (0.15)** *	**0.18 (0.04)** **
aAge	**	**	**	*	**	**
*SES (ref: Poorest Quart 1):*						
Poor (Quart 2)	**1.34 (0.18)** **	0.94 (0.15)	**1.21 (0.12)** *	**1.3 (0.13)** **	1.19 (0.22)	0.04 (0.05)
Rich (Quart 3)	1.15 (0.17)	1.2 (0.18)	0.9 (0.08)	**1.27 (0.13)** **	1.3 (0.24)	0.06 (0.05)
Richest (Quart 4)	1.02 (0.15)	0.9 (0.13)	**0.68 (0.07)** **	0.95 (0.11)	1.15 (0.22)	**−0.12 (0.06)** **
*Village (Ref: Kinango):*						
Jego	**2.97 (0.56)** **	**4.9 (1.5)** **	**0.35 (0.04)** **	**1.99 (0.27)** **	**9.58 (3.72)** **	**0.66 (0.13)** **
Magadzoni	**3.56 (0.38)** **	**2.05 (0.44)** *	**0.31 (0.04)** **	**1.48 (0.17)** **	**6.05 (2.38)** *	**0.44 (0.14)** **
Milalani	**2.1 (0.24)** **	**6.11 (1.17)** **	**1.36 (0.12)** **	**2.61 (0.28)** **	**44.7 (17.25)** **	**1.12 (0.13)** **
Nganja	0.66 (0.23)	**4.53 (0.95)** **	**1.52 (0.16)** **	**2.36 (0.29)** **	**46.53 (17.95)** **	**1.07 (0.14)** **
Vuga	1.17 (0.18)	**7.54 (1.4)** **	**0.47 (0.05)** **	**1.3 (0.14)** *	**11.36 (4.41)** **	**0.56 (0.14)** **
House inhabitants	**1.04 (0.02)** **	**1.03 (0.02)** *	**1.06 (0.02)** **	1.01 (0.02)	0.99 (0.03)	0.01 (0.01)
Use of bednet	**0.71 (0.08)** **	**1.27 (0.13)** *	-	-	-	**−0.23 (0.05)** **
Safe drinking water	-	-	-	-	**0.66 (0.11)** **	0.02 (0.04)
Presence of latrine	-	-	**0.7 (0.09)** **	**0.41 (0.04)** **	**0.55 (0.09)** **	**−0.31 (0.05)** *

Given its very low prevalence, ascariasis was excluded from the modeling analyses.

**Figure 3:**
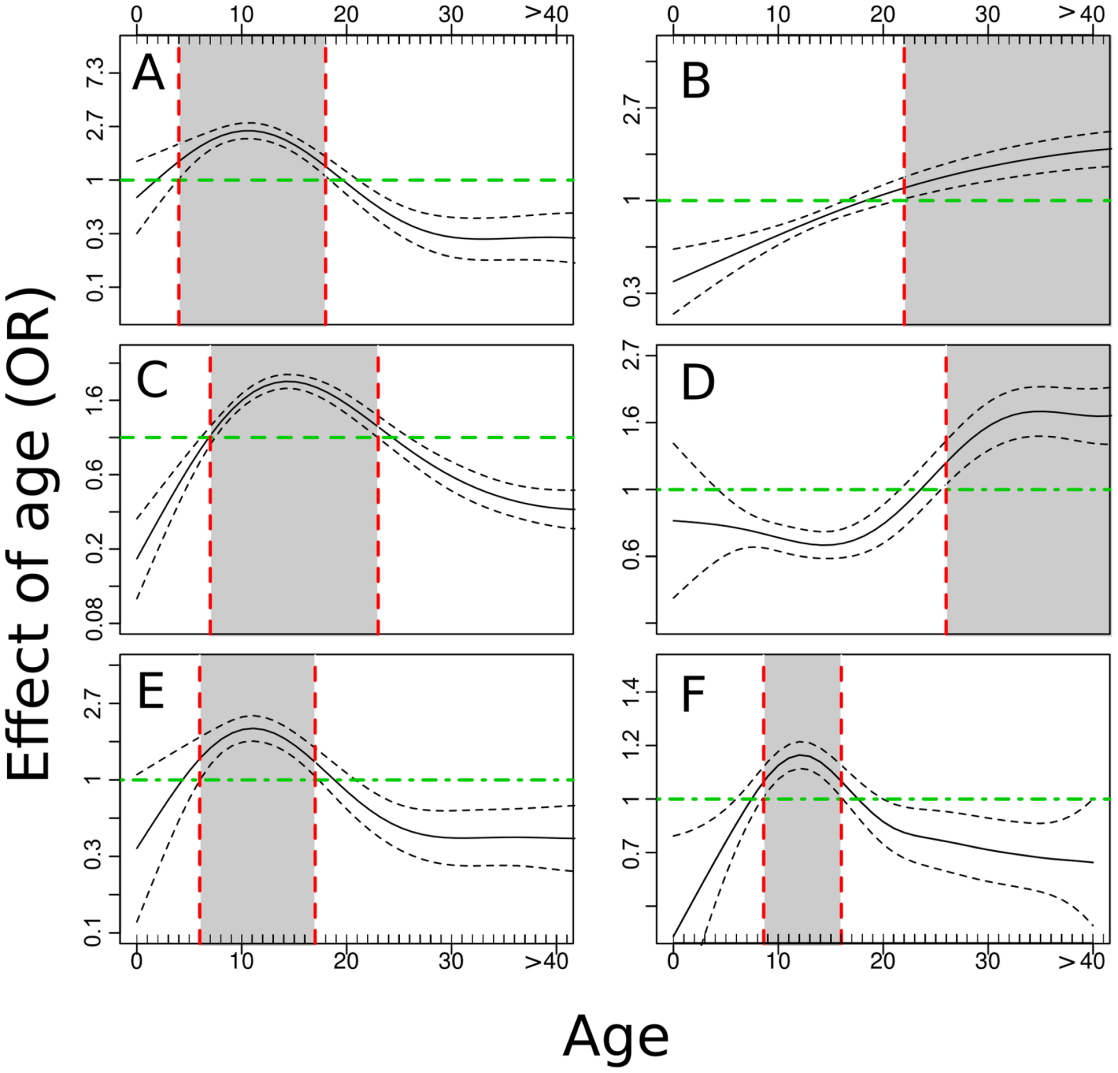
Association of age with single parasite infections and with poly-parasitism. Malaria (A), Filariasis (B), Schistosomiasis (C), Hookworm (D), Trichuriasis (E), Poly-parasitism (F). Gray areas in the graphs indicate age ranges associated with significantly higher levels of infection. The image include the mean value (solid lines) and 95% CI (dashed lines).

* = p<0.05;

** = p<0.01,

a Only the significance of the age factor is indicated; the smooth functions of age predictors are shown in Figure 3,

b adjusted Odds Ratio (OR) and Error Factor (EF),

c Value (SE).

**Figure 4:**
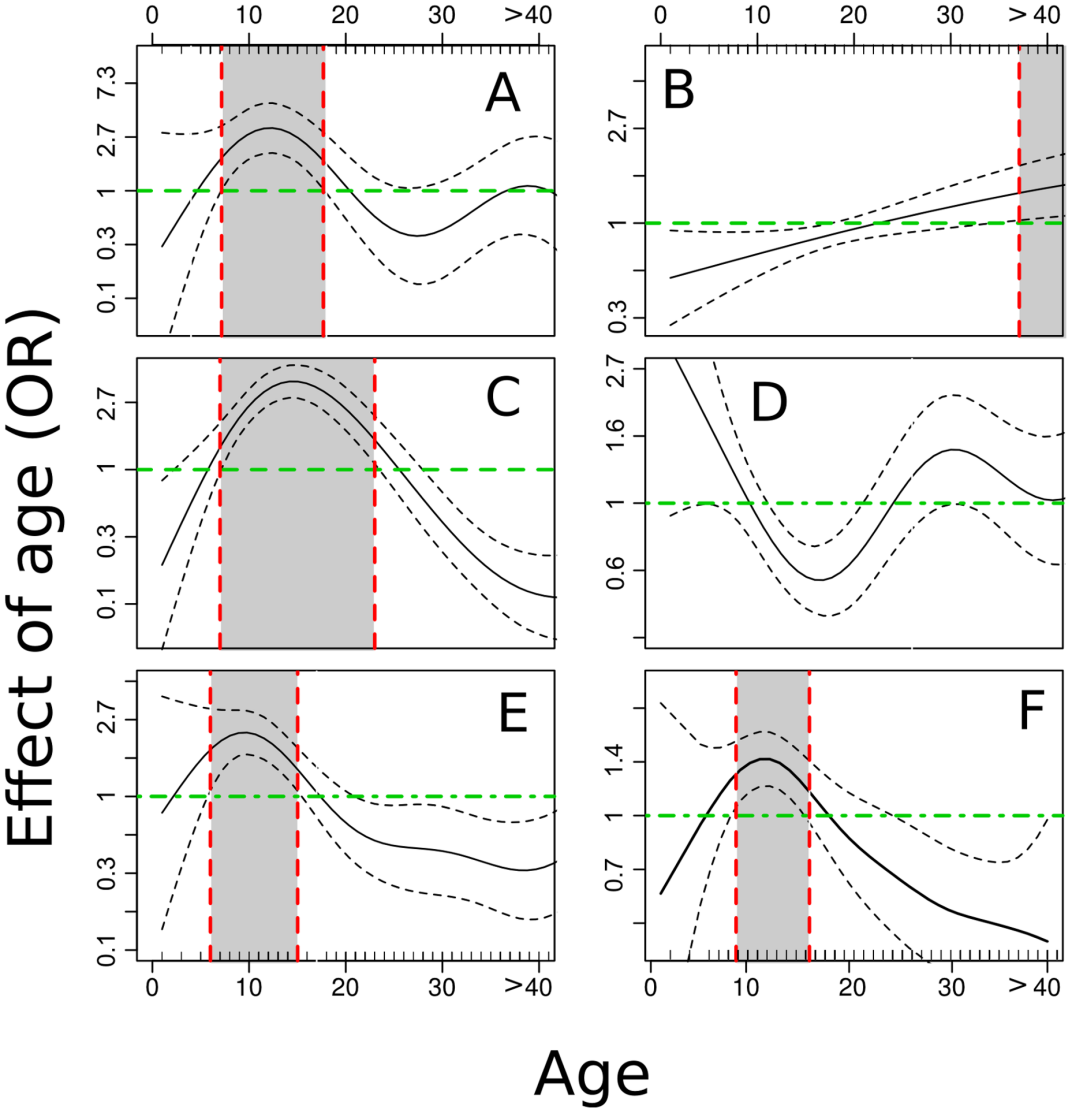
Association of age with single parasite infection and with poly-parasitism in Milalani. Malaria (A), Filariasis (B), Schistosomiasis (C), Hookworm (D), Trichuriasis (E), poly-parasitism (F). Gray areas in the graphs indicate age ranges associated with significantly higher levels of infection.

Age was an important factor affecting all single and co-infections (Figures 3 and 4, Figures S2 through S7 in Text S1). Aggregated prevalence data for all villages showed a reduction in malaria rates after age 19, an increase in filaria prevalence with age, a peak in *S. haematobium* prevalence for ages 10–19, an increase in hookworm infections with age, and a decline in *Trichuris* infections after age 19 (Figures S2–S6 in Text S1). In Milalani and Nganja, hookworm infections declined in the 10–19 age group before rising again in the older age groups. Overall, children and young adults were more likely to be infected with malaria parasites, *S. haematobium*, and *Trichuris*, while adults were more likely to be infected with filariasis and hookworms. Polyparasitism (two or more infections) was highest in the 10–19 age group. The smooth function, obtained from GAM, of age association with infection presence (Figure 3) provided a good fit to the results of the association of age with prevalence, with the exception of hookworms (Figure 3, panel D), which was less prevalent in ages 12–23. This overall effect was primarily a result of the age-patterns in Nganja and Milalani, where hookworm infections were most common.

Village and environmental factors were major independent correlates of infection. After controlling for demographic factors and SES, living in Kinango was protective with regard to several single infections and for co-infections. However, as shown by univariate analysis, living in Kinango was associated with increased risk of *S. haematobium* infection.

Results from the GAM applied to the entomological data (Table 4) showed no significant correlation between presence or number of collected mosquitoes and malaria or filariasis cases. However, not surprisingly, bednet use was protective against malaria.

**Table 4:** Association of abundance and presence of mosquito vectors with malaria and filaria infections in surveyed individuals.

	Malaria	Filariasis
Abundance^a^^,^ b:		
*An. gambiae*	0.01 (0.02)	0.01 (0.02)
*An. funestus*	−0.02 (0.01)	0.05 (0.04)
*Culex* spp.	-	0.01 (0.01)
Presence^c^:		
*An. gambiae*	0.57 (0.22)	0.73 (0.35)
*An. funestus*	1.38 (0.24)	1.04 (0.21)
*Culex* spp.	-	0.9 (0.29)

Predictors based on GAMs were adjusted for gender, age, education, location, SES, no. of house inhabitants, use of bednets, drinking water source, and sanitation.

a Number of collected mosquitos per house,

b Value (SE),

c Adjusted Odds Ratio (EF) for infection.

When the GAM and GAMM were applied to Milalani alone (Table 5), very few covariates (other than living in Milalani *per se*) were correlated with infection status. SES was significantly associated with the risk of being infected by any of the parasites only with lower risk of being infected by hookworms. The use of bednets was again a protective factor for malaria. Sanitation and access to safe source of drinking water were protective only against *Trichuris* and against poly-parasitism, which in Milalani was mostly the result of multiple helminthic infections. These results are consistent with the health status of the Milalani community, where the most common infections and co-infections were due to *S. haematobium* and STH. Age was again associated with single infections or co-infections (Figure 4), similar to the results obtained for the multi-village models.

**Table 5:** Predictor estimates obtained by GAMs per each parasite infection in Milalani.

Predictor	Malaria^b^	Filariasis^b^	Schistosomiasis^b^	Hookworm^b^	Trichuris^b^	Number of co-infections^c^
Sex: Male	1.27 (0.21)	1.02 (0.17)	1.04 (0.09)	1.23 (0.13)	1.09 (0.14)	0.12 (0.08)
aAge	**	**	**	**	**	**
*SES (ref: Poorest Quart 1):*						
Poor (Quart 2)	1.12 (0.24)	0.91 (0.19)	0.96 (0.17)	1.05 (0.15)	1.21 (0.21)	0.10 (0.11)
Rich (Quart 3)	1.01 (0.23)	0.78 (0.32)	0.9 (0.17)	1.21 (0.16)	1.28 (0.21)	0.12 (0.12)
Richest (Quart 4)	0.77 (0.18)	0.97 (0.05)	0.85 (0.17)	**0.48 (0.15)** **	1.39 (0.4)	−0.01 (0.06)
House inhabitants	1.15 (0.16)	1.26 (0.15)	0.97 (0.02)	1.03 (0.04)	0.99 (0.04)	0.01(0.01)
Use of bednet	**0.63 (0.16)** **	1.08 (0.28)	-	-	-	−0.11(0.08)
Safe drinking water	-	-	-	-	**0.6 (0.13)** *	−0.08 (0.11)
Presence of latrine	-	-	0.99 (0.12)	0.73 (0.17)	**0.59 (0.1)** **	**−0.32 (0.11)** *

Given the low number of infections with *Ascaris*, this parasite was not included in modeling analyses.

* = p<0.05;

** = p<0.01,

a Only significance of the age factor is reported; the smooth functions of age predictors are shown in Figure 4,

b OR (EF),

c Value (SE).

### Spatial analysis

**Figure 5:**
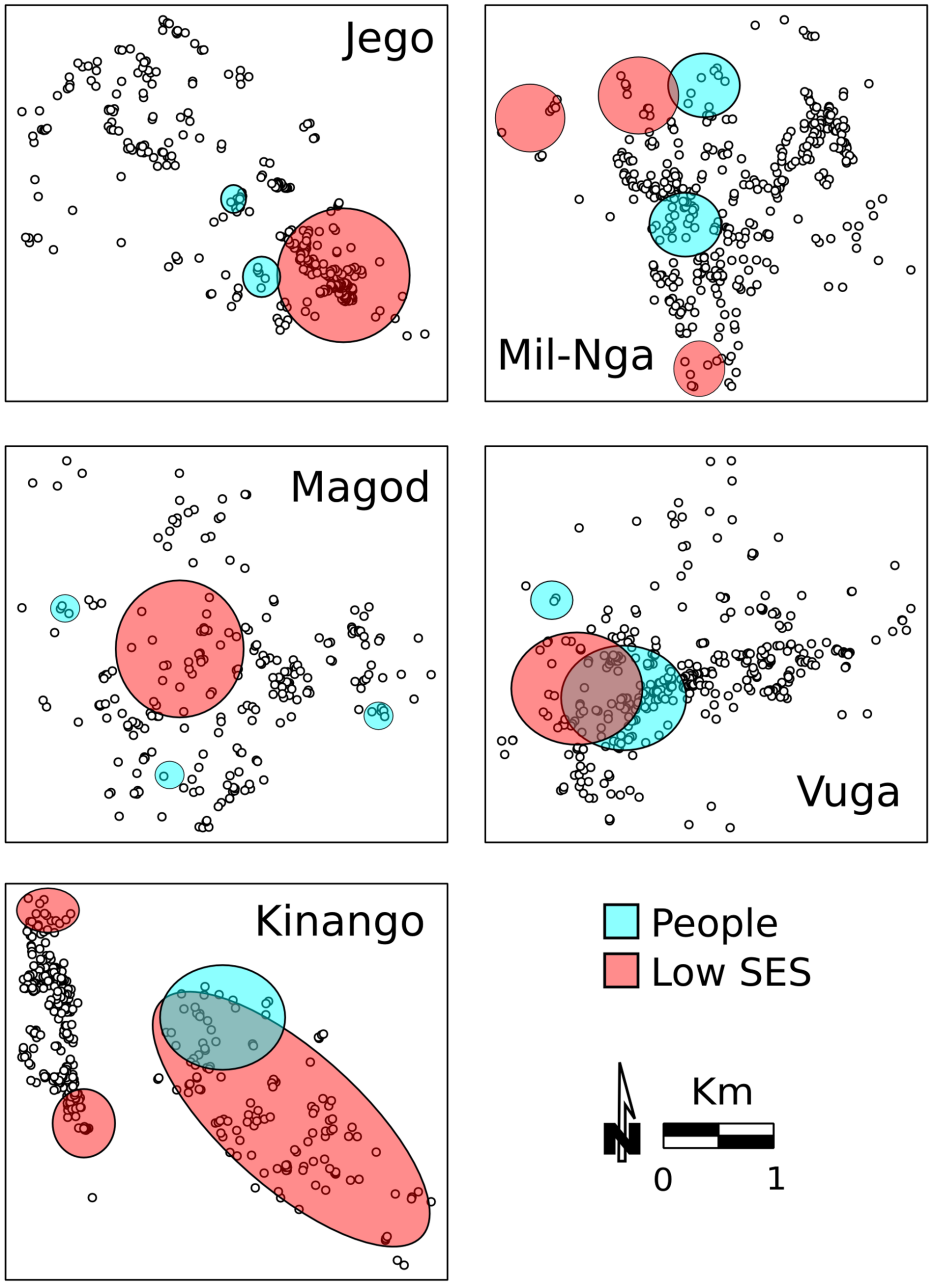
Occurrence of clusters of lower SES and of higher household density. Colored areas indicate significant clusters based on the G_i_*(d) test (p<0.05) of clusters of high levels of crowding and of low levels of SES.

Results based on the G_i_*(d) test showed that high density households and lower income were clustered in all villages (Figure 5). In Vuga and Kinango, the clustering pattern of both density and low income overlapped. Clusters of high household density were often located near the main road (Figure 5, Figure S1 in Text S1). In contrast, people with lower SES were usually clustered away from the main roads (Figure 5, Figure S1 in Text S1). Low-income houses were clustered closer to sites that were suitable for snail hosts of *S. haematobium* (Figure 5 and Figure S1 in Text S1).

**Figure 6:**
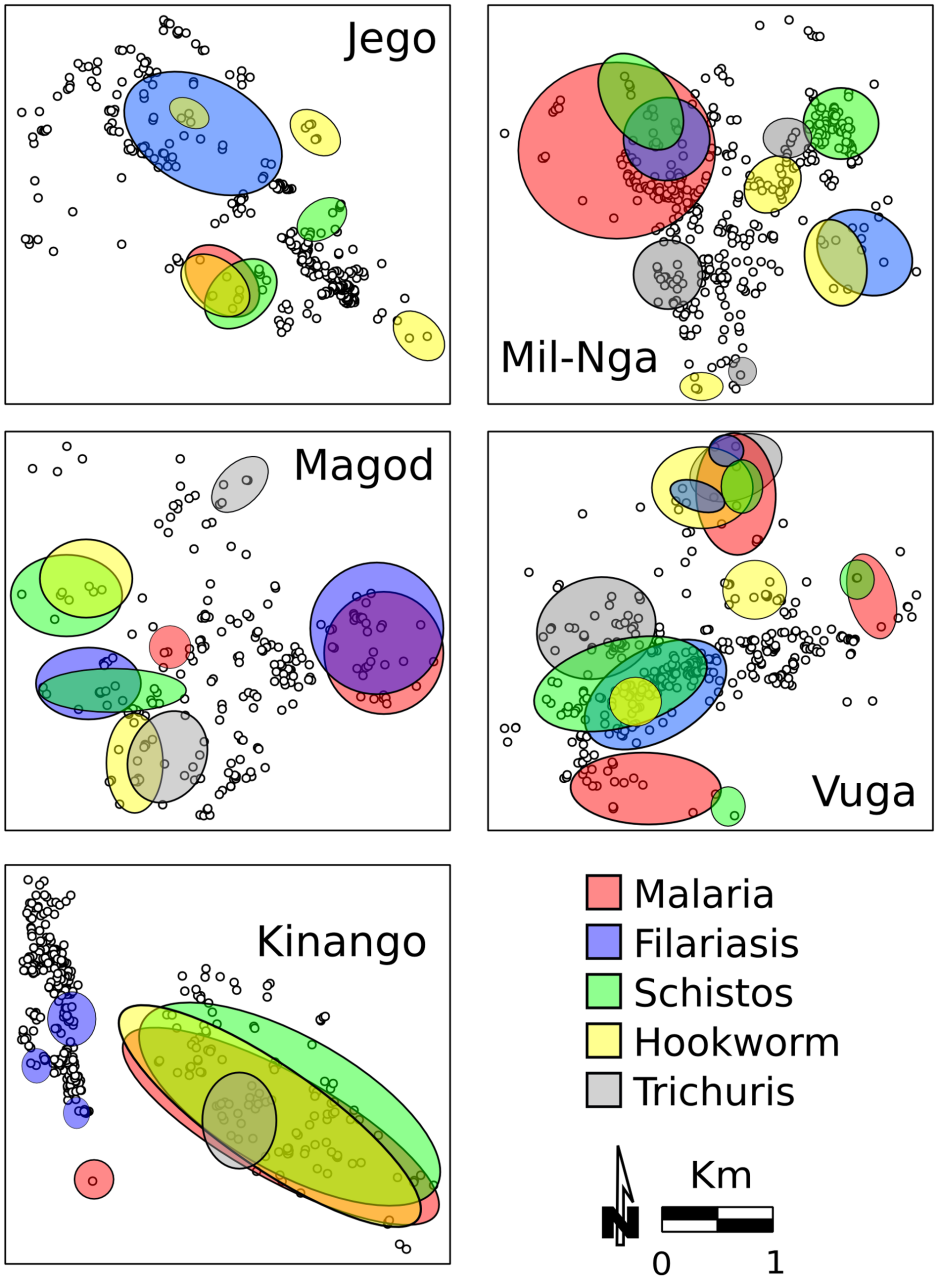
Occurrence of clusters for each parasite infection. Colored areas indicate significant hot-spots based on the G_i_*(d) test (p<0.05).

**Figure 7:**
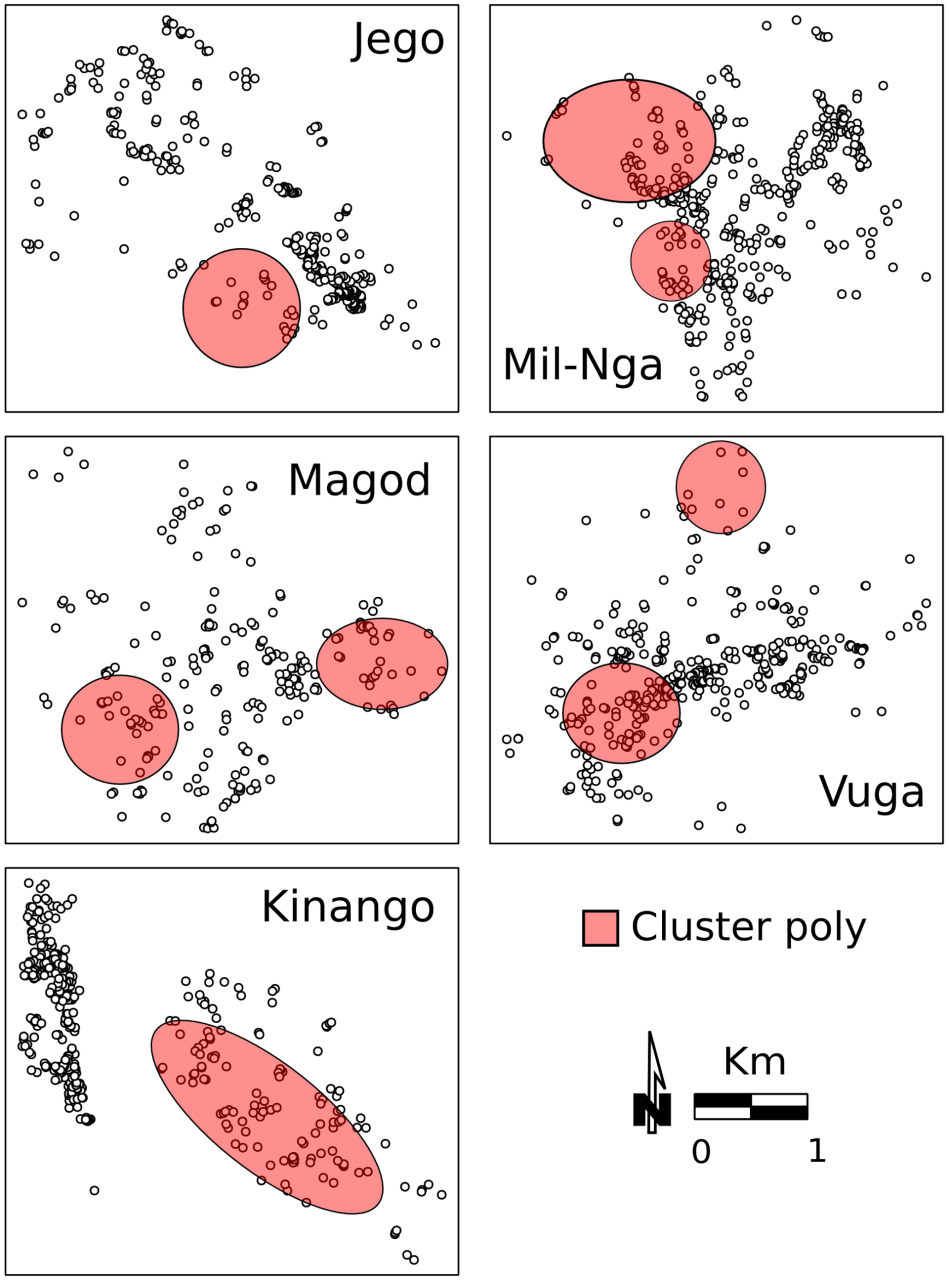
Occurrence of clusters for poly-parasitism. Colored areas significant hot-spots based on the G_i_*(d) test (p<0.05). The analysis is based on the prevalence of co-infections (any combination) recorded in each household.

The spatial distribution of prevalence of single parasitic infections and of poly-parasitism in each village was significantly clustered (Figures 6 and 7). Hot spots of diseases were not confined to a particular area in the villages, but, rather, overlapped each other (G_i_*(d) test, p<0.05). Malaria and schistosomiasis hot-spots co-occurred in the same locations in all villages except for Magodzoni. In Milalani and Jego there was only one malaria cluster per village, whereas the highest number of discrete malaria clusters (n = 3) was detected in Vuga. Clusters of the various helminthic infections overlapped in all villages, and were located near the main roads in all communities, with the exception of Kinango. In Kinango, almost all the spatial clusters of infections and co-infections were found in one area far from main roads and confined to the eastern part of the community. The exception was filariasis, which was clustered in the northwest part of the community. Not surprisingly, clusters of high levels of co-infection prevalence (Figure 7) were located where the highest numbers of single infection clusters were also found (Figure 6). Table 6 presents values and significance levels of Spearman's *ρ* used to describe correlation between disease hot spots and clusters of high population density and of lower SES.

**Table 6:** Results from Spearman's correlation test between co-occurrence of poly-parasitism clusters and clusters of low SES or of high population density.

	Malaria (HPD; LSES)	Filariasis (HPD; LSES)	Schistosomiasis (HPD; LSES)	Hookworm (HPD; LSES)	Trichuris (HPD; LSES)	Co-infection (HPD; LSES)
Jego	**0.19** **; −0.01	−0.06; −0.07	0.03; −0.04	−0.02;**−0.17** **	−0.01; −0.04	**0.16** **; **−0.14** **
Magodzoni	−0.02;**−0.15** **	−0.03;**−0.27** **	**0.16** **;**−0.16** **	−0.02; **−0.17** **	−0.01; −0.04	**0.17** **;**−0.14** **
Kinango	**0.25** **;**0.53** **	−0.05; 0.01	**0.27** **; **0.63** **	**0.35** **;**0.68** **	−0.06; **0.26** **	**0.16** **;**0.64** **
Milalani/Nganja	−0.05; 0.07	−0.03; −0.04	−0.04; **0.39** **	−0.03; 0.06	**0.12** *; 0.01	**0.11** *;**0.16** **
Vuga	**0.18** **; −0.01	−0.06; −0.07	0.03; −0.04	**0.17** **; −0.01	−0.05; −0.08	0.11;−0.05

This analysis was performed to determine whether households included in clusters of single or multiple infections were associated with presence of SES or high population density clusters.

HPD = high population density cluster; LSES = low SES cluster.

* = p<0.05;

** = p<0.01.

In several villages clusters of single infections and of co-infections were significantly correlated with the presence of clusters of high household density and of low SES (Table 6). Spatial correlation of single infection hot-spots with high household density clusters, when it was significant (with malaria in Jego, Kinango and Vuga, with *S. haematobium* in Magodzoni and Kinango, with hookworm infection in Kinango and Vuga, and with *Trichuris* in Milalani/Nganja), was always positive. Spatial clusters of households with lower income were significantly correlated with occurrence of infection. The correlation was either positive, mostly in Kinango (malaria, *S. haematobium*) and in Milalani/Nganja (hookworm and *Trichuris*), or negative (with malaria, filariasis, *S. haematobium*, and hookworms in Magadzoni, and with hookworm in Jego). The results were similar for co-infections, with significant positive correlations with high household density in all villages except for Vuga. Lower SES was positively correlated with poly-parasitism in Kinango and Milalani/Nganja and negatively in Jego and Magodzoni. Magodzoni was the only village in which there was no correlation between poly-parasitism clustering and either high household density or lower SES.

## Discussion

Simultaneous and sequential transmission of multiple parasites, and the resultant chronic/recurrent infections, are facts of life in many underdeveloped rural areas. They represent a significant, but often poorly recognized health and economic burden for affected populations [58], [59]. The chronic inflammatory process associated with long-term parasitism contributes to anemia and undernutrition [60], [61], [62] which, in turn, can lead to growth stunting, poor school performance [63], [64], poor work productivity [6], and continued poverty [6], [7]. Recently, a clear interest in integrated parasite control systems that can simultaneously target multiple NTDs is emerging. These national and international programs aim to create control systems based on knowledge from epidemiological analyses, such as the present study, that are performed to investigate the dynamics of multi-parasite transmission [18], [58], [65], [66], [67], [68].

Our study was focused on analyzing co-infections by several parasites and identifying factors associated with increased risk of polyparasitism. Our findings demonstrate that most infections have common risk factors (i.e., sanitation, SES, age), which increase the risk of co-infections for inhabitants of specific communities. Similar results have been showed by studies performed to investigate co-infection of malaria and hookworm in schoolchildren in coastal Kenya [17]. However, we have also shown that risk factors are not the same in each community. This may be associated with differences in environmental and population characteristics recorded in the villages. Integrating data from demographic, socio-economic, and behavioral surveys with spatial pattern of disease occurrence, we did not identify a particular risk area where all infections were clustered; rather, we were able to highlight co-infection hot-spots. We saw that individuals in the 8–16 age group were at high risk of exposure to malaria, schistosomiasis, and *Trichuris*, but not for filariasis and hookworms, which mostly affected adults.

Infection status of villages involved in our study was consistent with findings reported in previous studies performed in coastal Kenya [69], [70]. In this region, malaria, schistosomiasis, and STH are widespread throughout many communities affecting a high portion of population. Our results indicate that the most common infection was with STH, of which hookworm showed the highest prevalence. In our communities *A. lumbricoides* was rarely detected with an overall prevalence of 0.3%. It is well documented that this parasite infects only a few individuals in coastal Kenya, but that in other parts of Southern Kenya it reaches a prevalence of ∼20% [69], [70].

Prevalence of single and multiple infections were heterogeneous between the communities comprising this study. This coarse spatial pattern was associated with elevation, climatic, environmental and SES factors which affect diffusion and persistence of parasites [14], [18], [70], [71]. Similar spatial heterogeneity (at a larger scale) was highlighted by an extensive study examining co-infections across East Africa [17]. On a finer scale, within communities, our spatial analyses detected hot spots for each of the parasitic infections that we studied. Similar spatial heterogeneity at household level has also been reported for schistosomiasis in Kenya [1], [40], malaria in Mali [72], filariasis in Tanzania [73], and STH in Brazil and Panamá [14], [71]. We recorded co-infection clusters more often in those locations where several hot-spots of single infections overlapped, emphasizing the increased risk of polyparasitism where increased risk for individual infections is locally combined. In some villages, clusters of malaria, filariasis, and schistosomiasis overlapped or occurred near each other. These co-occurring clusters were found near aquatic habitats favorable for *Anopheles* mosquitoes and snails. Similarly, Mboera, et al. have reported that, in Tanzania, children living in proximity to rice fields are infected or co-infected with malaria and helminths more often than children living in dry areas [74].

We found a significantly higher prevalence of double infections than the rate predicted by the local prevalence of each individual parasite. This result could be due to a synergic effect of common risk factors (e.g., SES, sanitation) and parasite spatial distribution as shown by our analyses. Previous studies have also shown that the prevalence of co-infection prevalence could be increased by the interaction between helminths and *P. falciparum* [16], [33]. However, there are still conflicting reports on this topic, and the mechanisms underlying this possible interaction are still not completely understood [75].

Absence of spatial autocorrelation in residuals of each model showed that variables used to perform the analysis describe well the spatial pattern of the mono and co-infections. This result indicates that the spatial heterogeneity at village level is related with characteristics of households and their inhabitants. Previous studies have also shown that environmental factors are less likely than demographic and socioeconomic condition to capture spatial pattern of infection and co-infections at village scale [16], [33].

In our study, the two communities of Nganja and Milalani were contiguous and did not show a significant difference in prevalence of parasite infections and co-infection, with the exception of malaria. Many fewer *Anopheles* spp. females were collected in Nganja than in Milalani, and this difference could be explained by the presence of more larval sites in Milalani and the short dispersal distance of vector mosquitoes [76]. However, we did not find a significant correlation between mosquito abundance (or presence) and malaria or filarial infections. These results were likely affected by the limitations of the methods used to collect mosquitoes—based on our long-term surveillance for the present study, we previously detailed [77] that when abundance of mosquitoes is low, it is difficult to obtain a representative sample of local mosquito populations using classical sampling methods.

We found a marked relationship between urbanization, socio-economic development, and STH infections. Kinango, Vuga, and Magodzoni had lower STH prevalence compared to the other villages. These three communities averaged higher SES levels compared with the communities in the southern part of study area. Prevalence of STH in a community has been shown to be negatively correlated with the number of houses with a dirt floor, and positively associated with lack of access to good sanitation [14], [71], both of which impact parasite contact with humans and their spread in human environment. We found both single infections and co-infections to be associated with SES. These findings are consistent with results showed by similar studies investigating helminth and malaria mono and co-infection in sub-Saharan Africa [17], [33]. People who were classified as poor had higher risk of being infected with multiple infections. Within a community, the association of SES with disease prevalence was observed in Kinango's spatial pattern of infection: with the exception of filariasis, all single and co-infections were clustered in the less developed area of Kinango where a hot-spot of low SES was detected. In contrast, when we applied GAMs to the Milalani data community, there was no strong association between SES and parasite prevalence (with the exception of hookworm prevalence which was significantly lower among those with the highest SES). Milalani is a poor rural community, where the range in SES subdivision is less marked than in other communities. Such a limited association of SES with STH prevalence was also reported from Indian rural communities where SES did not vary significantly [78].

Children and young adults under age 20 were at higher risk for single infections and co-infections, with the exception of filariasis, which was more often detected in people over 20. A mass drug administration (MDA) had been performed in the study area in 2003 as part of the National Programme for Elimination of Lymphatic Filariasis (NPELF) [70], consisting of treatment with diethylcarbamazine citrate (DEC) and albendazole, which also has a de-worming effect for STH. This prior administration of albendazole may also have reduced the prevalence of hookworms in Milalani and Nganja residents, where prevalence was lowest in the 10–19 age group. Following drug administration, re-infection with *Trichuris* and *Ascaris* is rapid (less than one year to reach pre-treatment prevalence), but is longer for hookworm [79], which may explain why we did not find a similar likely effect of NPELF on *Trichuris* infection. School-based de-worming campaigns were performed in our study area once a year during 2005, 2008, and 2011. This national program had a less coverage than 2003 campaign. However, the treatment frequency adopted during the last campaigns did not have an important impact on long term prevalence and intensity of helminths in coastal Kenya [80].

Although children are at highest risk for most infections and co-infections, surveillance and control strategies need to target both children and adults both to reduce transmission and improve health status [69]. The challenge of polyparasitism is increasingly recognized as manifested by: 1) increasing appreciation of the health and social burden of chronic/recurrent infections [59], [81], [82]; 2) more sensitive diagnostics indicating that concurrent polyparasitism is much more prevalent than previously thought [18], [27]; and 3) new inexpensive approaches to treatment and transmission control becoming increasingly more accessible [58], [83], [84]. Until recently, conventional wisdom about parasites has been that light parasitic infections are mostly ‘asymptomatic’–meaning that they do not provoke symptoms that require medical attention [85]. However, new studies of immunopathology of infection and chronic disease formation [61], [62], [86], [87], indicate that the presence, as well as the intensity of infection, drives morbidity due to infection [7]. Under-recognized ‘subtle’ morbidities such as malnutrition, anemia, and poor school performance have been shown to be significant correlates of individual helminthic or protozoan infection [6], [59], [61], [62], [64], and concern is growing about the combined health effects of multiple concurrent parasite infections [67]. This is an important issue to take in consideration in coastal Kenya, where ∼50% of our study population was positive for at least one parasite and ∼20% were burdened with co-infections. These estimates are also bound to be underestimates since our parasitological methods (egg detection) are less than optimal diagnostic tools. It is possible, though, that this high parasitic burden has a major impact on the health status of the low-income populations, and limits the potential development of the region. The combined impact of these endemic infections has not been well studied, and may prove to have additive or more complex non-linear interactive effects. Consequently, optimal control strategies may require local reduction of both *transmission* (preventing infection) and *disease manifestations* through the integrated targeting in concert of one, some, or all of these parasitic infections. A key challenge for reducing transmission of these infections is the diversity in exposure and transmission routes, across multiple levels, including spatially and temporally. In this scenario, the results of our study should prove quite helpful in designing an integrated drug distribution plan for coastal Kenya. As previously shown [68], more cost-effective integrated systems can be impacted by increasing knowledge about the total infectious burden of target population. This should be achievable through the use of detailed disease mapping [68]. The prediction maps of co-distribution of NTDs in the literature were often developed using data collected for a specific demographic group such as schoolchildren from few schools in a specific district [10], [11], [17]. However, these data do not give a full picture of the actual health status of the population which should be targeted by a national control program. Our study highlighted how prevalence of single and multiple infections differed between age groups. We also pointed out that infection and co-infection prevalence of each village was not well represented by the overall parasitic prevalence. Our findings point to the need for applying the appropriate spatial scale and sampling strategy when designing and planning a survey system, and are especially relevant when drawing NTDs maps for the planning of effective MDAs in specific territories.

In terms of strengths and limitations, our study benefitted from the spatially diverse and long-term data that underlie it, from our familiarity and established relations with the communities that comprise the study population and with the study area. Our long-term association and rapport with the communities provided us with the local support necessary to enroll and collect the extensive data necessary for such an encompassing study. Our study is unique in the integration of environmental, demographic, socio-economic risk factors and entomological data with a broad parasitological outcomes. Our study also benefitted from the successful application of up to date spatial and multivariate techniques, such as spatial clustering, MCA, GAM, GAMM and a range of non-parametric tests. Like most field-based populations studies, we have encountered variable response rate in the different communities and lower rates of participation by adult males, which may have biased our results. Even with our more sensitive detection techniques, some infections have been missed, and, as a result, we could not always separate the relative contribution of the different risk factors for infection and co-infection. In particular, the environmental factors of distance from the sea, elevation and rainfall were highly correlated and their separate role could not be assessed. Given our very large database and our sophisticated yet cautious analytical approach, we are confident with regard to the significance of our findings and their implications.

### Conclusion

We have shown how several protozoan and helminthic parasites are widespread in southern coastal Kenya. In villages with high prevalence of helminthic infections (schistosomiasis and STH) and malaria, co-infections were clustered in areas where environmental and human risk factors (*i.e.*, low SES, poor sanitation, age, and presence of water bodies) came together to enhance the combined transmission of several parasites. The challenge of polyparasitism is increasingly recognized through i) our increasing appreciation of the health and social burden of chronic/recurrent infections [59], [81], [82]; ii) more sensitive diagnostics, which indicate that concurrent polyparasitism is much more prevalent than previously thought [18], [27]; and iii) new inexpensive approaches to treatment and transmission control that are increasingly more accessible [58], [83], [84]. We underline the heterogeneities among and within communities that need to be taken into account when planning appropriate surveillance and control strategies that target polyparasitism. Although children are at highest risk for most infections and co-infections, surveillance and control strategies need to target children and adults, both to reduce transmission and to reduce parasite-related disease burden [69].

## Supporting Information

Checklist S1 **STROBE checklist.**
(PDF)

Text S1 **The file includes supplementary information about variables used to create an SES index, prevalence of single and double parasitic infections by village, poly-parasitism prevalence in the six villages in coastal Kenya, spatial pattern of households in the study area, and health status per age group of study participants at village level.**
(DOC)
