# Supplementary material for: Cross-sectional Study of the Burden of Vector-Borne and Soil-Transmitted Polyparasitism in Rural Communities of Coast Province, Kenya
Source: PLoS Negl Trop Dis. 2014 Jul 24;8(7):e2992. doi: 10.1371/journal.pntd.0002992 (PMC4109907; doi:10.1371/journal.pntd.0002992)
Supplement: Checklist S1 — STROBE checklist. (PDF) [file pntd.0002992.s001.pdf]

STROBE Statement—Checklist of items that should be included in reports of ***cross-sectional studies***

	Item No	Recommendation
**Title and abstract**	1	(*a*) Indicate the study's design with a commonly used term in the title or the abstract Line 1
		(*b*) Provide in the abstract an informative and balanced summary of what was done and what was found Lines 18-42
**Introduction**		
Background/rationale	2	Explain the scientific background and rationale for the investigation being reported Page 4
Objectives	3	State specific objectives, including any prespecified hypotheses Lines 70-74, 103-112
**Methods**		
Study design	4	Present key elements of study design early in the paper Page 6
Setting	5	Describe the setting, locations, and relevant dates, including periods of recruitment, exposure, follow-up, and data collection Lines 124-133
Participants	6	(*a*) Give the eligibility criteria, and the sources and methods of selection of participants Lines 118-121
Variables	7	Clearly define all outcomes, exposures, predictors, potential confounders, and effect modifiers. Give diagnostic criteria, if applicable Line 139-Infection; Line 151-Mosquito counts; Line 170-SES
Data sources/ measurement	8*	For each variable of interest, give sources of data and details of methods of assessment (measurement). Describe comparability of assessment methods if there is more than one group
Bias	9	Describe any efforts to address potential sources of bias Line 158; Line 201
Study size	10	Explain how the study size was arrived at Lines 125-136
Quantitative variables	11	Explain how quantitative variables were handled in the analyses. If applicable, describe which groupings were chosen and why
Statistical methods	12	(*a*) Describe all statistical methods, including those used to control for confounding Line: 178-MCA; 189-General Additive Model; 192-GAMM; 203-212-Spatial Analysis; 215-Fisher's least significant difference; 217-Wilcoxon signed-rank test; 218 Sperman's correlation test
		(*b*) Describe any methods used to examine subgroups and interactions Lines 188-200
		(*c*) Explain how missing data were addressed Line 228
		(*d*) If applicable, describe analytical methods taking account of sampling strategy
		(*e*) Describe any sensitivity analyses- N/A
**Results**		
Participants	13*	(a) Report numbers of individuals at each stage of study—eg numbers potentially eligible, examined for eligibility, confirmed eligible, included in the study, completing follow-up, and analysed Table 1
		(b) Give reasons for non-participation at each stage
		(c) Consider use of a flow diagram- N/A
Descriptive data	14*	(a) Give characteristics of study participants (eg demographic, clinical, social) and information on exposures and potential confounders Line 232, Table 1S.

		(b) Indicate number of participants with missing data for each variable of interest N/A
Outcome data	15*	Report numbers of outcome events or summary measures Figure 1
Main results	16	(*a*) Give unadjusted estimates and, if applicable, confounder-adjusted estimates and their precision (eg, 95% confidence interval). Make clear which confounders were adjusted for and why they were included
		(*b*) Report category boundaries when continuous variables were categorized
		(*c*) If relevant, consider translating estimates of relative risk into absolute risk for a meaningful time period- N/A
Other analyses	17	Report other analyses done—eg analyses of subgroups and interactions, and sensitivity analyses Lines 285-321; 323-355
**Discussion**		
Key results	18	Summarise key results with reference to study objectives Lines 367-377
Limitations	19	Discuss limitations of the study, taking into account sources of potential bias or imprecision. Discuss both direction and magnitude of any potential bias Lines 487-501;
Interpretation	20	Give a cautious overall interpretation of results considering objectives, limitations, multiplicity of analyses, results from similar studies, and other relevant evidence Lines 496-499
Generalisability	21	Discuss the generalisability (external validity) of the study results 507-515
**Other information**		
Funding	22	Give the source of funding and the role of the funders for the present study and, if applicable, for the original study on which the present article is based This is loaded in the PNTD site and appears in the final article after the author list.

*Give information separately for exposed and unexposed groups.

Note: An Explanation and Elaboration article discusses each checklist item and gives methodological background and published examples of transparent reporting. The STROBE checklist is best used in conjunction with this article (freely available on the Web sites of PLoS Medicine at http://www.plosmedicine.org/, Annals of Internal Medicine at http://www.annals.org/, and Epidemiology at http://www.epidem.com/). Information on the STROBE Initiative is available at www.strobe-statement.org.
