# Supplementary material for: Cross-sectional Study of the Burden of Vector-Borne and Soil-Transmitted Polyparasitism in Rural Communities of Coast Province, Kenya
Source: PLoS Negl Trop Dis. 2014 Jul 24;8(7):e2992. doi: 10.1371/journal.pntd.0002992 (PMC4109907; doi:10.1371/journal.pntd.0002992)
Supplement: Text S1 — The file includes supplementary information about variables used to create an SES index, prevalence of single and double parasitic infections by village, poly-parasitism prevalence in the six villages in coastal Kenya, spatial pattern of households in the study area, and health status per age group of study participants at village level. (DOC) [file pntd.0002992.s002.doc]

**Supplementary Information**

**Table S1. Variables used to create an SES index and for applying Multiple Correspondenc**e Analysis (MCA)

| SES factors | Jego | Kinango | Magodzoni | Milalani | Nganja | Vuga |
| --- | --- | --- | --- | --- | --- | --- |
|  |  |  |  |  |  |  |
| *Cooking method (%):* |  |  |  |  |  |  |
| Charcoal | 5.3 | 32.4 | 1.8 | 6.9 | 12.6 | 1.5 |
| Electricity | 0.2 | 0.5 | 0 | 0 | 0 | 0 |
| Firewood | 90.4 | 62.9 | 97.4 | 87.7 | 83.8 | 96.3 |
| Gas | 0 | 0.5 | 0 | 0 | 0 | 0.7 |
| Paraffin | 1.2 | 3.3 | 0.5 | 0.6 | 0.6 | 0.7 |
|  |  |  |  |  |  |  |
| *Source of drinking water (%):* |  |  |  |  |  |  |
| Borehole | 0.4 | 0 | 0 | 65.7 | 49.7 | 4.4 |
| Dam | 12.1 | 0.3 | 57.4 | 0 | 0 | 0 |
| House | 1.8 | 17.9 | 0.3 | 0.3 | 6 | 2.2 |
| Public source | 48.1 | 77.9 | 38.8 | 34 | 44.3 | 82.5 |
| Rain | 0 | 1.3 | 2.1 | 0 | 0 | 0.5 |
| River | 35.6 | 2.6 | 1.6 | 0 | 0 | 10.3 |
|  |  |  |  |  |  |  |
| *Light source (%):* |  |  |  |  |  |  |
| Candles | 0 | 0 | 0 | 0 | 3 | 0 |
| Electricity | 4.3 | 16.7 | 0.3 | 6 | 2.4 | 0.7 |
| Lamp | 89.2 | 61.6 | 79.3 | 79.4 | 73.1 | 87.2 |
| Lantern | 5.7 | 20.2 | 19.9 | 14 | 19.8 | 9.6 |
| Solar | 0.6 | 1.5 | 0.3 | 0.6 | 0.6 | 2 |
| Wood | 0 | 0 | 0.3 | 0 | 0 | 0 |
|  |  |  |  |  |  |  |
| *Ownership (%):* |  |  |  |  |  |  |
| Own land | 53.6 | 66.8 | 92.8 | 92.9 | 82 | 86.7 |
| Own house | 91.6 | 78.4 | 96.4 | 84 | 86.2 | 93.1 |
| Telephone | 39.1 | 57.7 | 43.7 | 36.6 | 40.1 | 32.5 |
| Radio | 39.7 | 50.7 | 65.9 | 49.7 | 68.9 | 51 |
| Television | 2.5 | 17.9 | 10.6 | 4 | 16.2 | 11.8 |
| Bicycle | 38.7 | 22.9 | 43.2 | 36 | 44.9 | 9.9 |
| Motor vehicle | 1.6 | 4.7 | 5.7 | 2.3 | 3 | 1.2 |
| Servant | 2.9 | 6.7 | 1 | 0.3 | 1.8 | 1.2 |
|  |  |  |  |  |  |  |
| *Latrine (%):* |  |  |  |  |  |  |
| Inside house | 6.3 | 14.6 | 2.8 | 12.9 | 22.2 | 16.7 |
| No latrine | 85.9 | 45.9 | 65.6 | 76.9 | 61.1 | 32 |
| Outside (without water) | 7 | 37.6 | 31.3 | 10 | 10.2 | 50.5 |
| Flushing toilet | 1.2 | 9.1 | 1.0 | 2.0 | 6.0 | 2.2 |
|  |  |  |  |  |  |  |
| Mean number of rooms per household | 2.7 | 3.2 | 3.5 | 3.6 | 4.0 | 3.7 |
|  |  |  |  |  |  |  |
| *Floor material (%):* |  |  |  |  |  |  |
| Cement | 8.8 | 39.5 | 19.4 | 20.6 | 41.9 | 21.2 |
| Soil | 90.8 | 60.5 | 80.4 | 76.3 | 55.1 | 78.8 |
|  |  |  |  |  |  |  |
| *Roof material (%):* |  |  |  |  |  |  |
| Iron | 11.9 | 64.6 | 14 | 13.7 | 24 | 22.4 |
| Natural | 87.9 | 35.4 | 86 | 84 | 75.4 | 77.6 |
|  |  |  |  |  |  |  |

**Table S2. Prevalence of single and double parasitic infections by village**.
 * prevalence significantly (p<0.05) lower than expected; ** prevalence significantly (p<0.05) higher than expected

|  | Jego | Kinango | Magodzoni | Milalani | Nganja | Vuga | Overall |
| --- | --- | --- | --- | --- | --- | --- | --- |
| *Single infection:* |  |  |  |  |  |  |  |
|  |  |  |  |  |  |  |  |
| Malaria (MA) | 16.3%  (14.2-18.5%) | 6.5%  (5.1-8.1%) | 18.3%  (15.6-21.3%) | 12.4%  (10.1-14.9%) | 3.8%  (2.3-5.8%) | 7.1%  (5.8-8.6%) | 10.7%  (9.9-11.6%) |
| Filariasis (FI) | 10.6%  (8.9-12.5%) | 2.1%  (1.3-3.1%) | 4.5%  (3.1-6.2%) | 12.8%  (10.5-15.3%) | 9.8%  (7.4-12.6%) | 15.1%  (13.2-17.1%) | 9.3%  (8.6-10.1%) |
| Schistosomiasis (SC) | 16.6%  (14.5-18.9%) | 33.5%  (30.8-36.3%) | 15.6%  (13.1-18.4%) | 41.6%  (38.1-45.2%) | 41.6%  (37.4-45.9%) | 18.1%  (16-20.2%) | 26%  (24.8-27.1%) |
| Hookworm (HK) | 28.8%  (26.3-31.5%) | 13.3%  (11.4-15.4%) | 19.5%  (16.7-22.6%) | 31.8%  (28.6-35.2%) | 25.8%  (22.1-29.7%) | 15%  (13.1-17%) | 21.4%  (20.3-22.5%) |
| *T. trichuria* (TR) | 7.9%  (6.4-9.6%) | 1.8%  (1.1-2.8%) | 3.7%  (2.4-5.3%) | 27.3%  (24.2-30.6%) | 22%  (18.6-25.8%) | 7.3%  (5.9-8.8%) | 9.9%  (9.2-10.7%) |
| *A. lumbricoides* (AS) | 0.3%  (0-0.3%) | 0%  (0-0.3%) | 0.1%  (0-0.5%) | 0.6%  (0-0.5%) | 0.2%  (0-0.7%) | 0.3%  (0-0.3%) | 0.3%  (0-0.1%) |
|  |  |  |  |  |  |  |  |
| *Coinfection* |  |  |  |  |  |  |  |
|  |  |  |  |  |  |  |  |
| MA-FI | 0.9% [1.7]  (0.5-1.7%) | 0% [0.1]  (0-0.3%) | 0.3% [0.8]  (0.1-0.9%) | 1% [1.58]  (0.45-2.02%) | 0.4% [0.3]  (0.1-1.3%) | 1% [1.1]  (0.5-1.6%) | **0.6% [1]***  **(0.4-0.8%)** |
| MA-SC | **4% [2.7]****  **(2.9-5.3%)** | **3.9% [2.1]****  **(2.9-5.2%)** | **5.3% [2.8]****  **(3.7-7.1%)** | **9% [5.1]****  **(7.1-11.2%)** | 2.6% [1.5]  (1.4-4.3%) | 1.7% [1.2]  (1.1-2.5%) | **4.2% [2.7]****  **(3.6-4.7%)** |
| MA-HK | 5.5% [4.7]  (4.3-6.9%) | 1.5% [0.9]  (0.7-2.4%) | 3% [3.5]  (1.8-4.4%) | 4.6% [3.9]  (3.2-6.3%) | 1.5% [0.9]  (0.6-2.9%) | 0.9% [1.1]  (0.5-1.5%) | **2.8% [2.3]****  **(2.3-3.2%)** |
| MA- TR | 2% [1.2]  (1.2-2.9%) | 0.3% [0.1]  (0.1-0.8%) | 0.8%[0.6]  (0.3-1.7%) | 4.4% [3.3]  (3.1-6.1%) | 1.7% [0.8]  (0.7-3.1%) | 0.4% [0.5]  (0.1-0.8%) | **1.4% [1.1]****  **(1.1-1.7%)** |
| MA- AS | 0% [0.1]  (0-0.3%) | 0% [0.1]  (0-0.3%) | 0% [0.1]  (0-0.5%) | 0% [0.1]  (0-0.4%) | 0% [0.1]  (0-0.6%) | 0% [0.1]  (0-0.3%) | 0% [0.1]  (0-0.1%) |
| FI-SC | 1% [1.7]  (0.5-1.7%) | 0.8% [0.7]  (0.4-1.5%) | 1.4% [0.7]  (0.6-2.4%) | 4.6% [5.3]  (3.2-6.3%) | 4.9% [4.1]  (3.2-7.1%) | **3.8% [2.7]****  **(2.8-4.9%)** | 2.5%[2.4]  (2.1-2.9%) |
| FI-HK | **4.5% [3.1]****  **(3.4-5.8%)** | 0.4% [0.3]  (0.1-1.1%) | 1.1% [0.8]  (0.4-2.1%) | 5% [4.1]  (3.6-6.8%) | **4.1% [2.5]****  **(2.6-6.2%)** | 2.1% [2.2]  (1.4-3.1%) | **2.7% [2]****  **(2.31-3.17%)** |
| FI-TR | **0.2% [0.8]***  **(0.1-0.7%)** | 0% [0.1]  (0-0.3%) | 0% [0.1]  (0-0.5%) | 3.1% [3.4]  (1.9-4.5%) | 1.9% [2.2]  (0.9-3.4%) | 0.8% [1.1]  (0.4-1.5%) | 0.8% [0.9]  (0.6-1.1%) |
| FI- AS | 0% [0.1]  (0-0.3%) | 0% [0.1]  (0-0.3%) | 0% [0.1]  (0-0.5%) | 0% [0.1]  (0-0.4%) | 0% [0.1]  (0-0.7%) | 0% [0.1]  (0-0.3%) | 0% [0.1]  (0-0.1%) |
| SC-HK | 4.9% [4.8]  (3.8-6.3%) | 5.5% [4.5]  (4.3-7.1%) | **5% [3.1]****  **(3.5-6.8%)** | 12.5% [13.2]  (10.2-15.1%) | 10.4% [10.7]  (7.9-13.2%) | **3.7% [2.7]****  **(2.7-4.8%)** | **6.3% [5.5]****  **(5.6-6.9%)** |
| SC-TR | **2.2% [1.3]****  **(1.5-3.2%)** | 0.6% [0.6]  (0.2-1.2%) | 0.4% [0.5]  (0.1-1.1%) | **14.3% [11.3]****  **(11.9-16.9%)** | **12.4% [9.2]****  **(9.7-15.5%)** | 1.6% [1.3]  (0.9-2.4%) | **4.1% [2.5]****  **(3.6-4.6%)** |
| SC-AS | 0% [0.1]  (0-0.3%) | 0% [0.1]  (0-0.3%) | 0% [0.1]  (0-0.5%) | 0.3% [0.2]  (0.1-0.9%) | 0.2% [0.1]  (0-1.1%) | 0% [0.1]  (0-0.3%) | 0% [0.1]  (0-0.15%) |
| HK-TR | **3.5% [2.3]****  **(2.5-4.7%)** | 0.4% [0.2]  (0.1-1%) | 0.7% [0.7]  (0.2-1.5%) | **12.6% [8.7]****  **(10.4-15.2%)** | 7.3% [5.7]  (5.3-9.9%) | **1.9% [1.1]****  **(1.2-2.7%)** | **3.7% [2.1]****  **(3.2-4.2%)** |
| HK-AS | 0.1% [0.1]  (0-0.4%) | 0% [0.1]  (0-0.3%) | 0% [0.1]  (0-0.5%) | 0.1% [0.2]  (0-0.7%) | 0.2% [0.1]  (0-1.1%) | 0.1% [0.1]  (0-0.4%) | 0.1% [0.1]  (0.1-0.2%) |
| TR- AS | 0.2% [0.1]  (0.1-0.6%) | 0% [0.1]  (0-0.3%) | 0% [0.1]  (0-0.5%) | 0.4% [0.2]  (0.1-1.1%) | 0% [0.1]  (0-0.7%) | 0% [0.1]  (0-0.2%) | **0.1% [0.1]****  **(0.1-0.2%)** |
|  |  |  |  |  |  |  |  |

**Table S3.** Poly-parasitism prevalence in the six villages in costal Kenya

|  |  | No. infections (%) | | |  |  |
| --- | --- | --- | --- | --- | --- | --- |
| Village | 0 | 1 | 2 | 3 | >3 | Tot. |
|  |  |  |  |  |  |  |
| Jego | 514 (43.6%) | 434 (36.8%) | 181 (15.3%) | 46 (3.9%) | 4 (0.3%) | 1179 |
| Kinango | 625 (54.1%) | 421 (36.4%) | 88 (7.6%) | 20 (1.7%) | 1 (0.1%) | 1155 |
| Magodzoni | 400 (54.2%) | 232 (31.5%) | 93 (12.6%) | 11 (1.5%) | 1 (0.1%) | 737 |
| Milalani | 212 (27.3%) | 267 (34.4%) | 196 (25.3%) | 82 (10.5%) | 19 (2.4%) | 776 |
| Nganja | 184 (34.6%) | 191 (35.9%) | 118 (22.2%) | 31(5.8%) | 7 (1.3%) | 531 |
| Vuga | 700 (52.4%) | 461 (34.5%) | 150 (11.2%) | 20 (1.5%) | 4 (0.3%) | 1335 |
| Overall | 2635 (46.1%) | 2006 (35.1%) | 826 (14.5%) | 210 (3.7%) | 36 (0.6%) | 5713 |
|  |  |  |  |  |  |  |


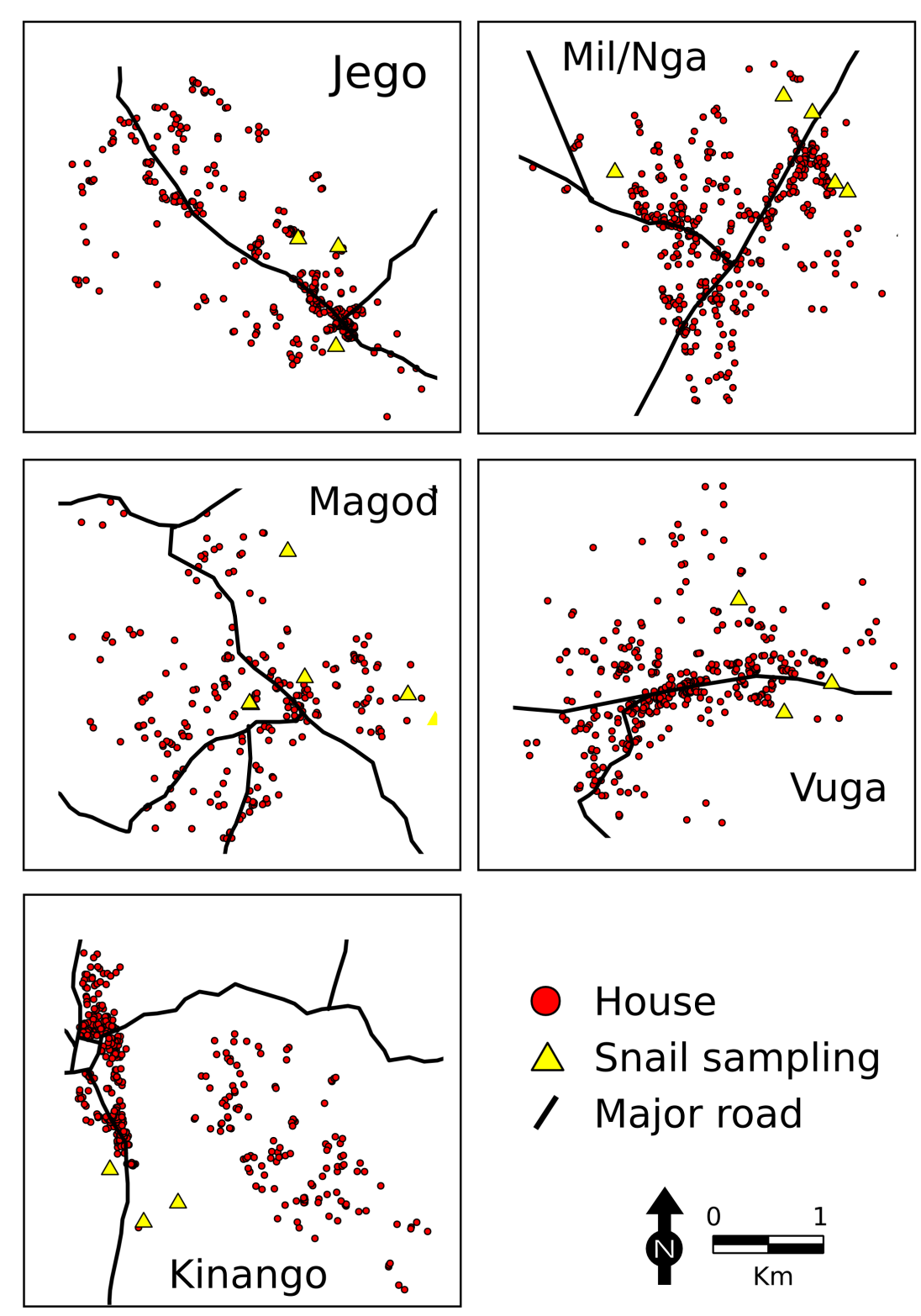


**Figure S1. Spatial pattern of households in the study area.** Main roads and snail sampling locations (part of the *S. haematobium* Study) are also shown.


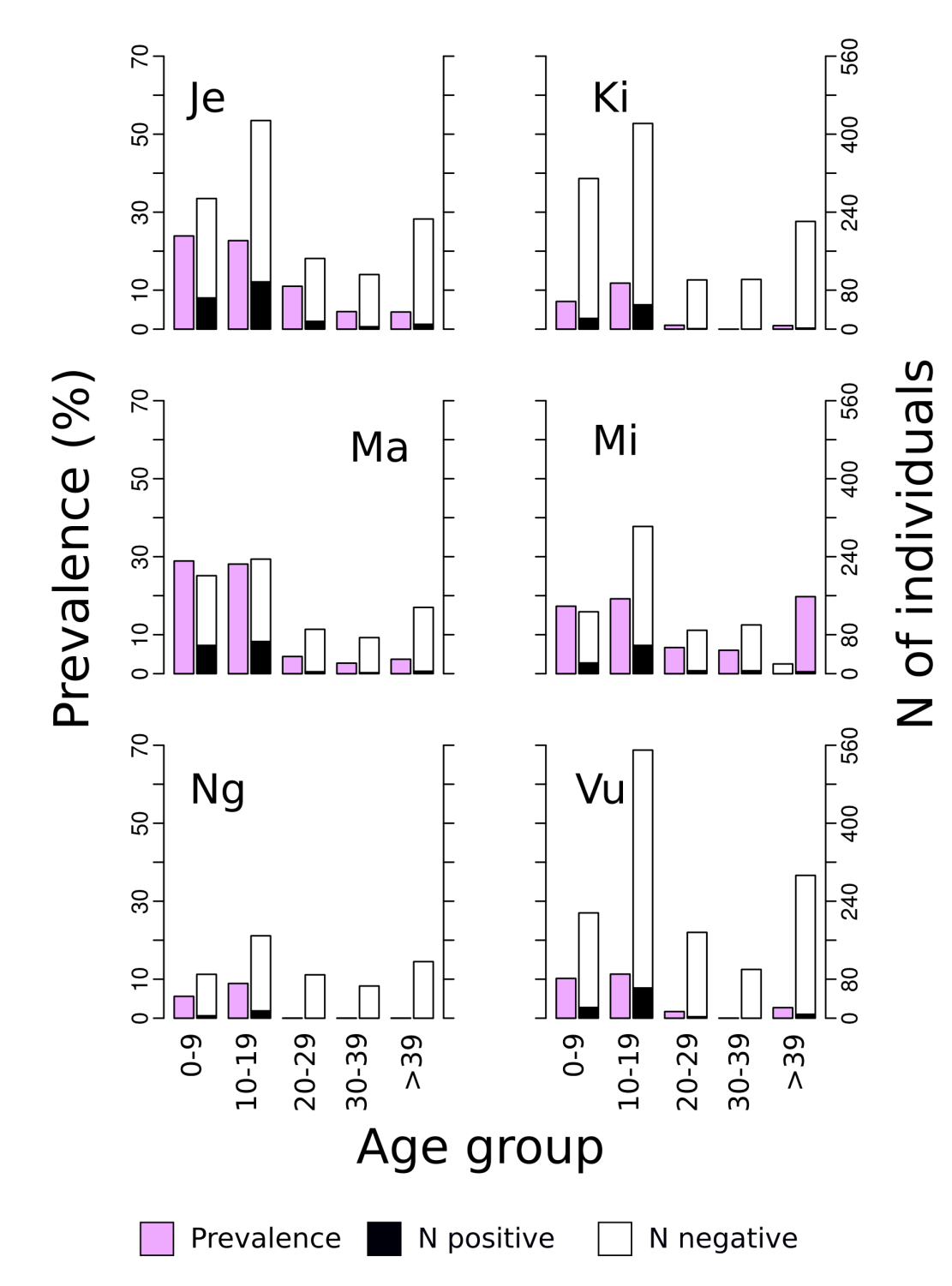


**Figure S2. Malaria infection status by age group at the village level**


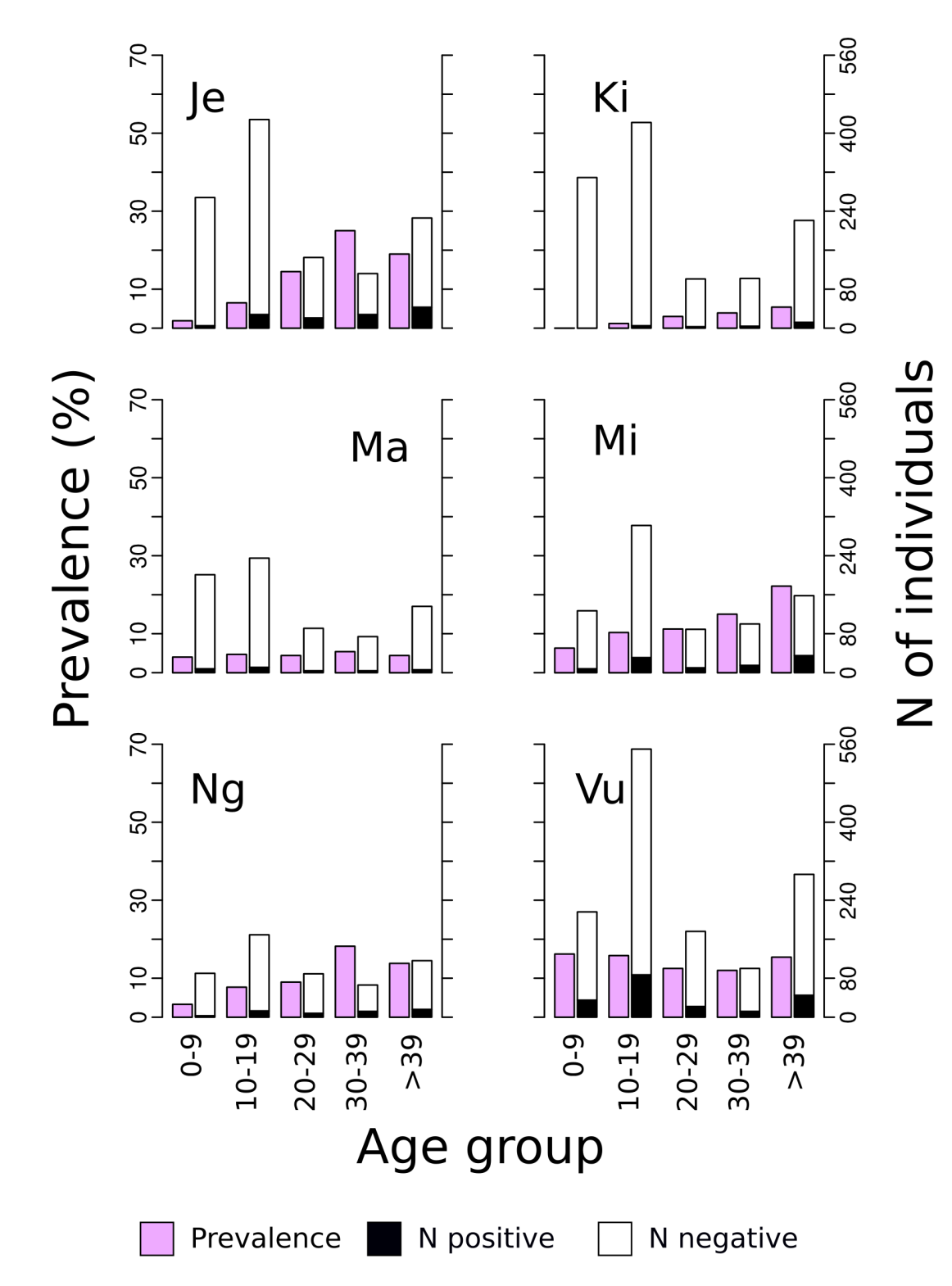


**Figure S3. Filariasis infection status by age group at the village level**


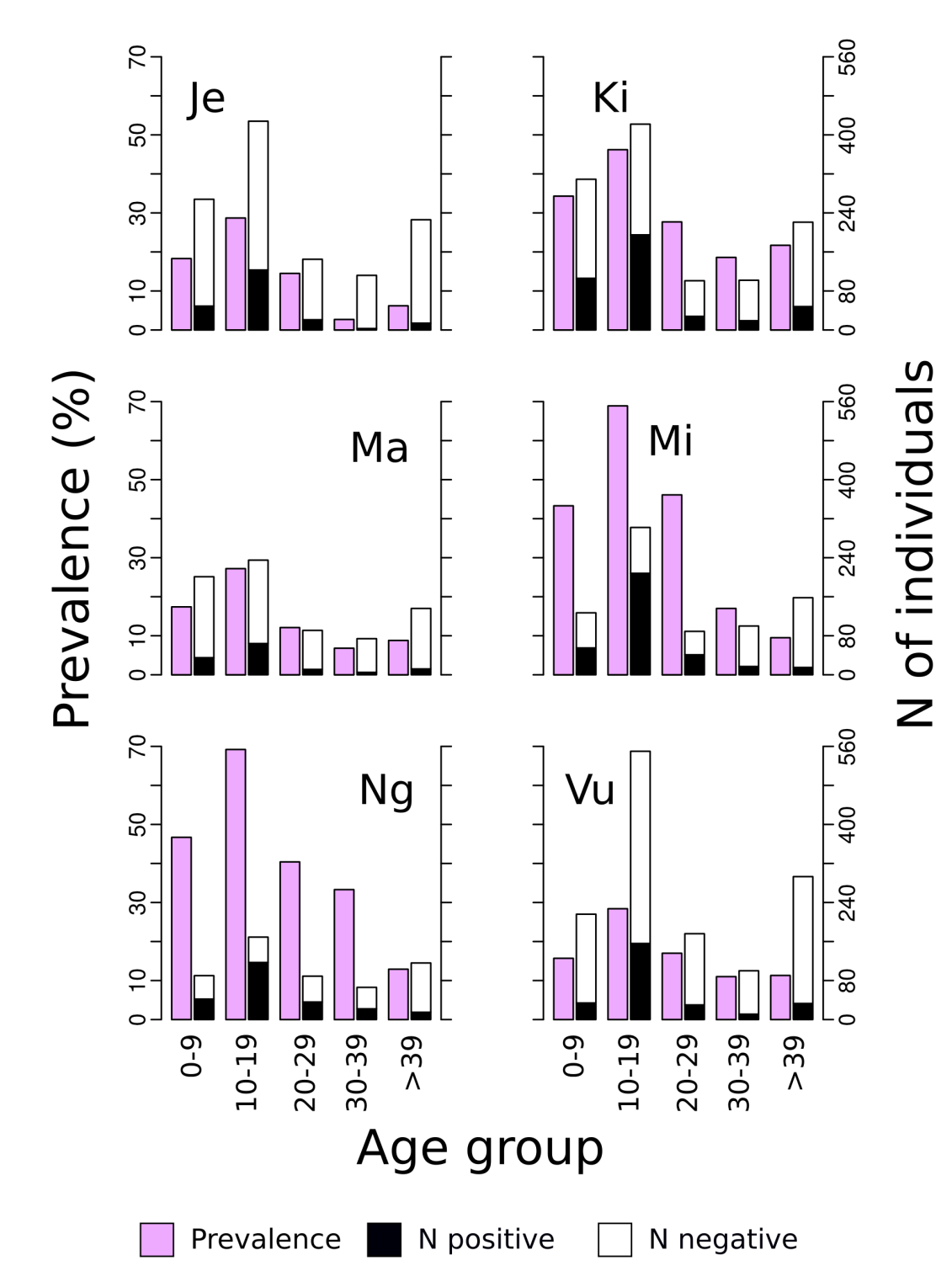


**Figure S4. Schistosomiasis infection status by age group at the village level**


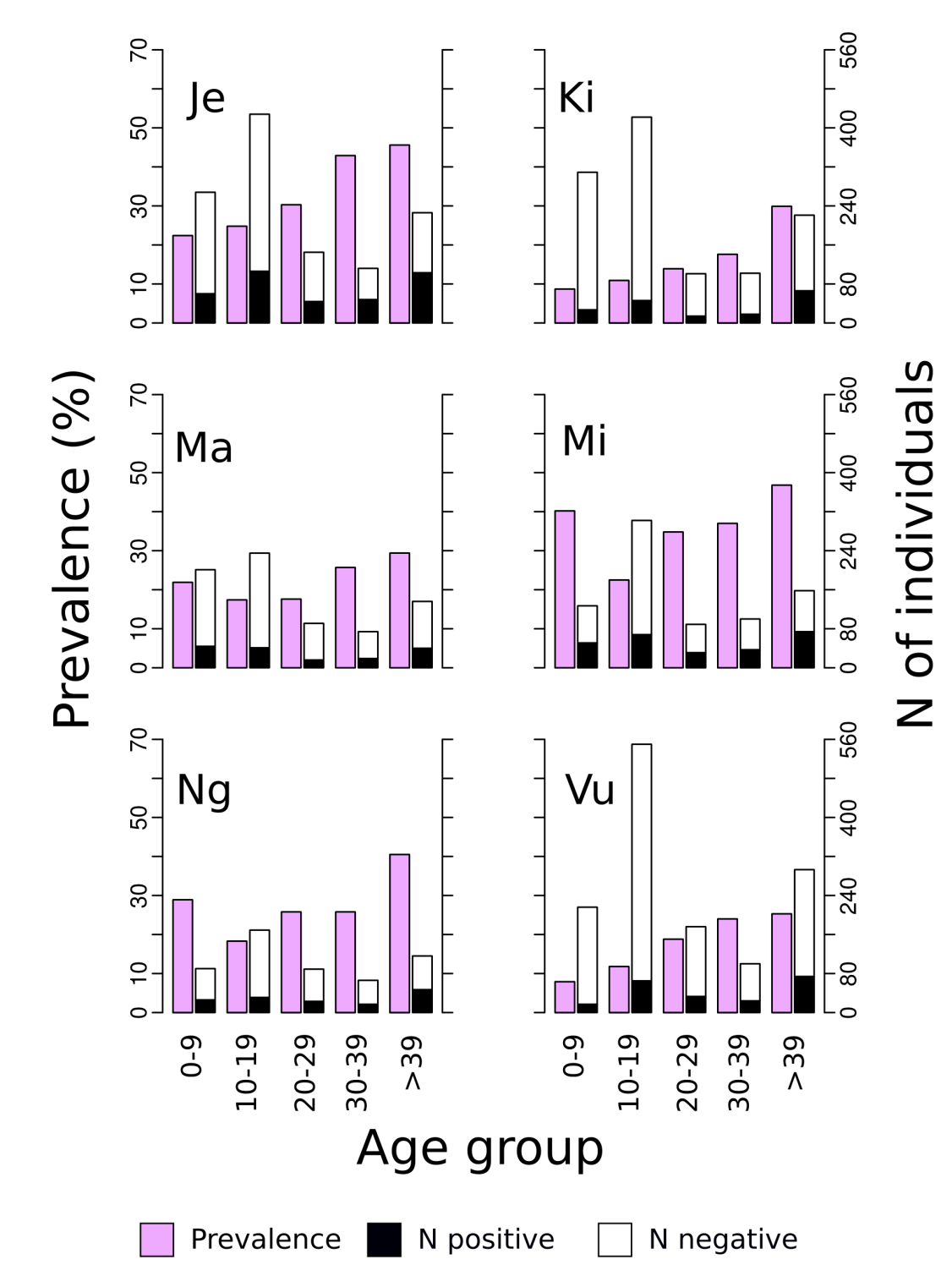


**Figure S5. Hookworm infection status per age group at the village level**


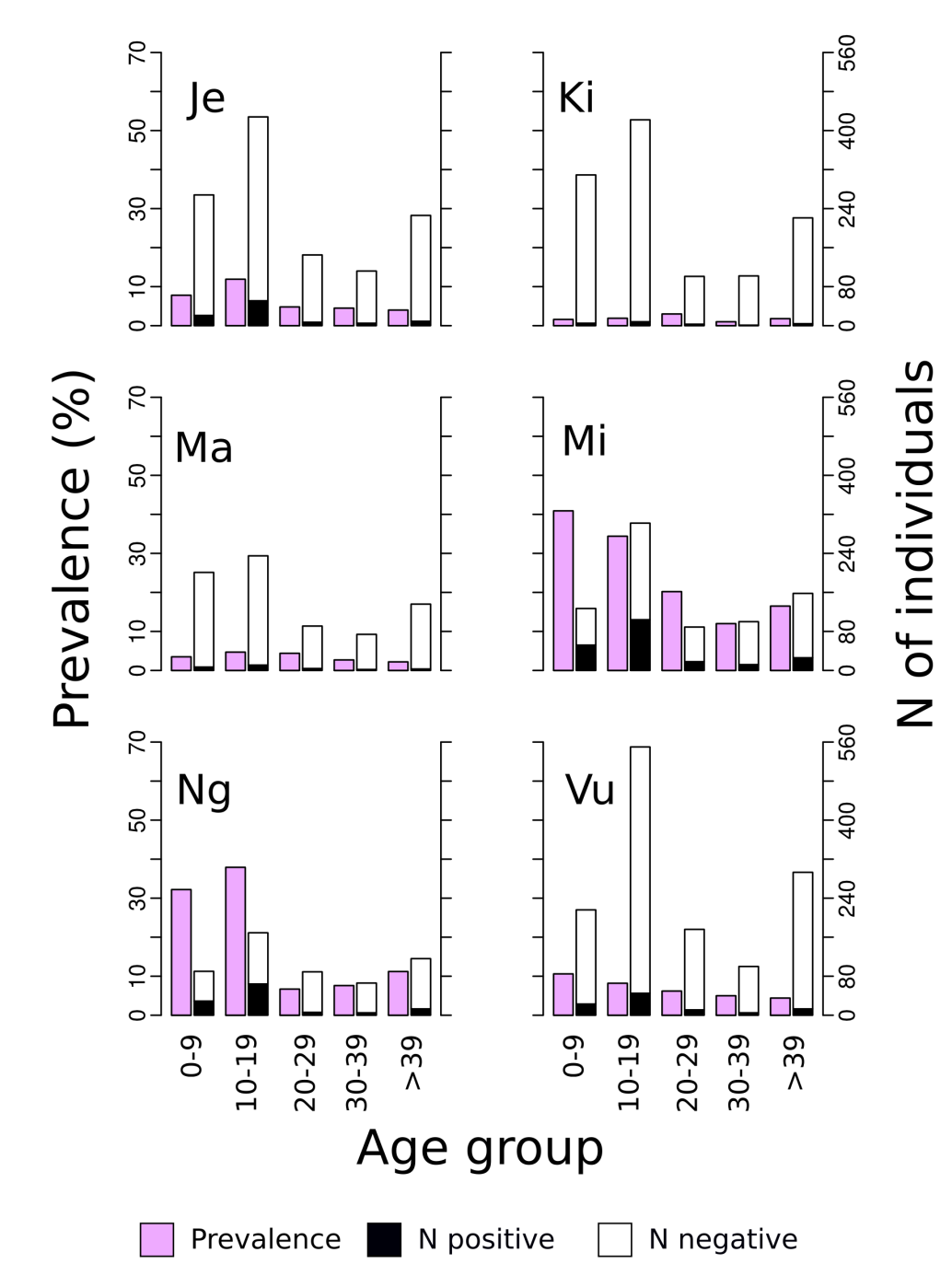


**Figure S6. *Trichuris* infection status by age group at the village level**


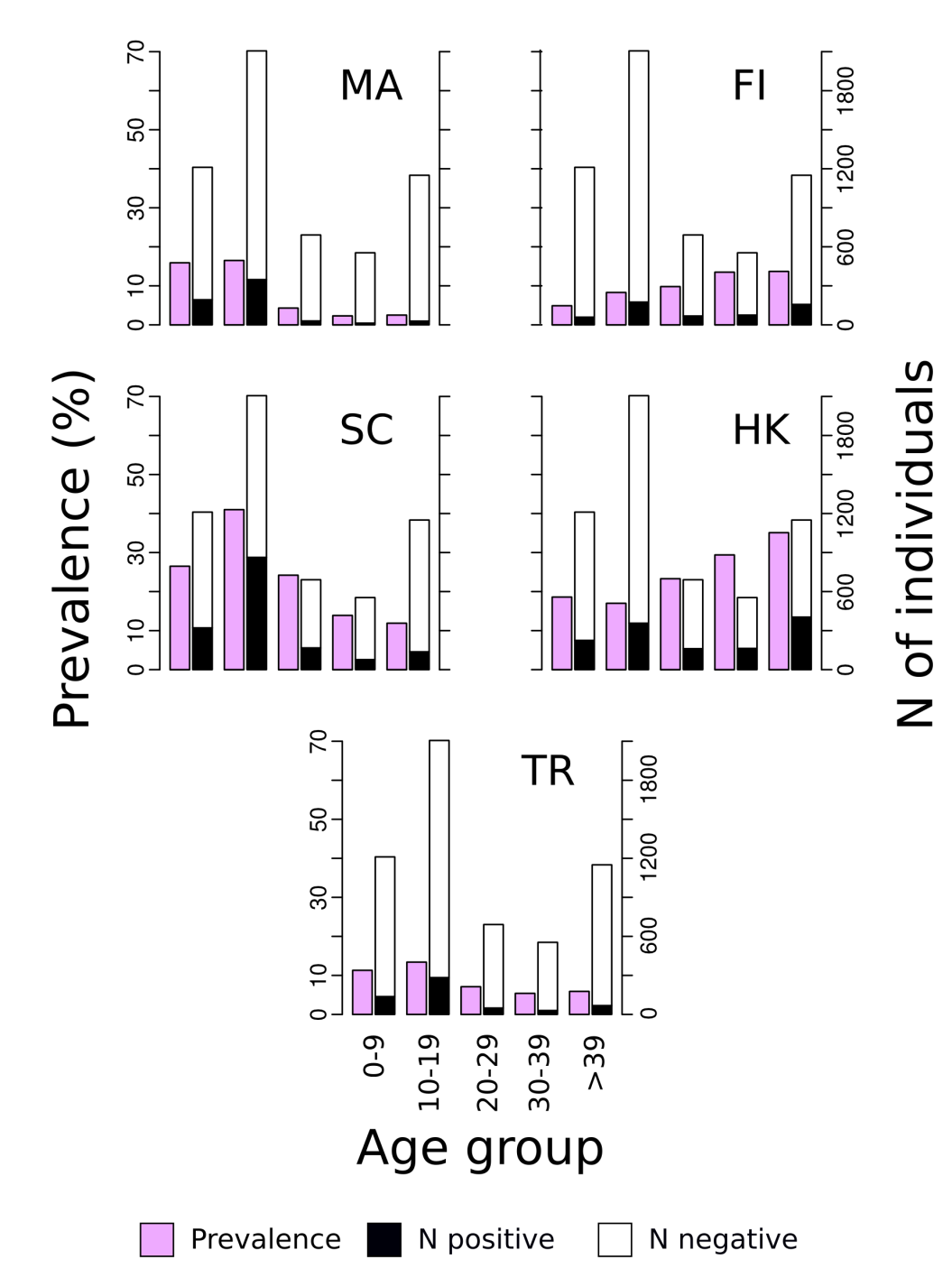


**Figure S7. Health status per age group of study participants.** Malaria (MA), Filariasis (FI), Schistosomiasis (SC), Hookworm (HK), and Trichuriasis (TR).
